# Ocean‐Wide Conservation Genomics of Blue Whales Suggest New Northern Hemisphere Subspecies

**DOI:** 10.1111/mec.17619

**Published:** 2024-12-17

**Authors:** Magnus Wolf, Menno J. de Jong, Axel Janke

**Affiliations:** ^1^ Senckenberg Biodiversity and Climate Research Centre (BiK‐F) Frankfurt am Main Germany; ^2^ Institute for Evolution and Biodiversity (IEB), University of Muenster Muenster Germany; ^3^ Institute for Ecology, Evolution and Diversity, Goethe University Frankfurt am Main Germany; ^4^ LOEWE‐Centre for Translational Biodiversity Genomics (TBG) Senckenberg Nature Research Society Frankfurt am Main Germany

**Keywords:** blue whales, conservation genomics, genetic diversity, population genomics, runs of homozygosity, subspecies

## Abstract

The blue whale is an endangered and globally distributed species of baleen whale with multiple described subspecies, including the morphologically and genetically distinct pygmy blue whale. North Atlantic and North Pacific populations, however, are currently regarded as a single subspecies despite being separated by continental land masses and acoustic call differences. To determine the degree of isolation among the Northern Hemisphere populations, 14 North Pacific and 6 Western Australian blue whale nuclear and mitochondrial genomes were sequenced and analysed together with 11 publicly available North Atlantic blue whale genomes. Population genomic analyses revealed distinctly differentiated clusters and limited genetic exchange among all three populations, indicating a high degree of isolation between the Northern Hemisphere populations. Nevertheless, the genomic and mitogenomic distances between all blue whale populations, including the Western Australian pygmy blue whale, are low when compared to other inter‐subspecies distances in cetaceans. Given that the Western Australian pygmy blue whale is an already recognised subspecies and further supported by previously reported acoustic differences, a proposal is made to treat the two Northern Hemisphere populations as separate subspecies, namely *Balaenoptera musculus musculus* (North Atlantic blue whale) and *Balaenoptera musculus sulfureus* (North Pacific blue whale). Furthermore, a first molecular viability assessment of all three populations not only found a generally high genomic diversity among blue whales but also a lack of alleles at low frequency, non‐neutral evolution and increased effects of inbreeding. This suggests a substantial anthropogenic impact on the genotypes of blue whales and calls for careful monitoring in future conservation plans.

## 1 Introduction

Conservation genomics aims at understanding the molecular dynamics within or between threatened populations to propose beneficial conservation plans (Frankham et al. 2017). Such projects usually aim to describe precise management units through population structure analyses (Andrews et al. 2018; Attard et al. 2018; Walters and Schwartz 2021) or to assess the genetic ‘viability’ by studying genetic diversity, effects of inbreeding and mutational load of severely reduced populations (Foote et al. 2021; von Seth et al. 2021; van der Valk et al. 2019). An important but often overlooked task is to resolve taxonomic uncertainties that might oversimplify conservation efforts. This could eventually misdirect these efforts to too small or too large groups of individuals and handicap the political process of developing laws and treaties meant to protect them (Mace 2004; Taylor et al. 2017).

Such genetic analyses are particularly important in cases where traditional means of observation are difficult, as in the case of threatened marine animals that are often highly mobile and widely distributed in the open sea, such as whales (Taylor et al. 2017). Although there is a growing body of whale conservation genetics literature (Archer et al. 2019; Attard et al. 2024; Foote et al. 2021; de Greef et al. 2022; Leslie and Morin 2018; Onoufriou et al. 2022; Wolf et al. 2022), studies that combine population structure analyses, genetic viability estimations and taxonomic re‐assessments are rare but needed given that marine species often exhibit both hidden population structures and subspeciation (Archer et al. 2019; Onoufriou et al. 2022; Taylor et al. 2017) and the molecular impact of anthropogenic interactions is often unknown, except in a few cases (Crossman, Fontaine, and Frasier 2023; de Greef et al. 2024; Wolf et al. 2022).

With maximum body lengths exceeding 30 m (Branch et al. 2007; McClain et al. 2015), the blue whale ( *Balaenoptera musculus* ) is the largest baleen whale (Mysticeti) and the largest species known to have ever existed on Earth (Sears and Perrin 2009). Blue whales are found in all major oceans and their distribution is only restricted by oligotrophic central oceanic regions that lack productive ocean dynamics such as upwelling and frontal meandering (Branch et al. 2007). Most individuals participate in seasonal migrations between productive feeding and warmer or safer breeding grounds that require them to travel long distances (Burtenshaw et al. 2004; Double et al. 2014). In some cases, individuals have been recorded travelling distances of up to 8000 km during one season (Hucke‐Gaete et al. 2018), showcasing their ability for trans‐oceanic migrations.

Several blue whale subspecies with different distributions, morphologies, acoustic and molecular patterns have been described (Perrin, Mead, and Brownell Jr 2009). The pygmy blue whale (*B. m. brevicauda*) is one of the smallest subspecies and was originally described by Ichihara in 1966 based on morphological features from individuals observed around Kerguelen Island in the southern East‐Indian Ocean (49° S, Ichihara 1966). While strongly supported by distinct morphological features such as shorter total body length, shorter baleen plates, ‘silvery’ coloration and potentially different body proportions, it remains uncertain, how the name *B. m. brevicauda* should be applied due to the unknown identity of the type specimen (Perrin, Mead, and Brownell Jr 2009). Nevertheless, whales from the southern Indian Ocean, especially in the east near the coast of Australia, form a morphologically, acoustically and genetically distinct clade which is commonly referred to as the ‘pygmy blue whale’ (Attard et al. 2024; Branch et al. 2007; Double et al. 2014; Stafford et al. 2011) and will be used synonymously throughout this study.

There is also a distinction between whales from the Southern Hemisphere around Antarctic waters (*B. m. intermedia*, Burmeister 1871–1872) and whales from the Northern Hemisphere (*B. m. musculus*, von Linné 1758) based on vague descriptions of body size differences (Tomilin 1946) and the expectation of limited gene flow across equatorial regions (Sears and Perrin 2009). As for the pygmy blue whale, the application of the Antarctic subspecies epithet is uncertain because the type specimen from the Río Luján near Buenos Aires could originate from either the *B. m. brevicauda* or the *B. m. intermedia* Southern Hemisphere subspecies (Perrin, Mead, and Brownell Jr 2009). Furthermore, there is an ongoing debate about whether southern East Pacific blue whales, distributed alongside the coast of Chile, form a subspecies distinct from *B. m. brevicauda* and *B. m. intermedia* (Branch et al. 2007; LeDuc et al. 2007; Pastene, Acevedo, and Branch 2019). However, blue whales from the Northern Hemisphere are usually not divided into further subspecies, despite recognised differences in acoustics (McDonald, Mesnick, and Hildebrand 2006; Mellinger and Clark 2003) and expected limited gene flow given the separation by continental landmasses.

Like all baleen whales, the number of all blue whales were dramatically reduced by commercial hunting in the 20th century (Aguilar and Borrell 2022; Tønnessen and Johnsen 1982). The Southern Hemisphere Antarctic blue whale is one of the most extreme examples of baleen whale exploitation and estimates using Bayesian models fitted onto sighting data suggest a reduction to 1% of its original abundance (Branch, Matsuoka, and Miyashita 2004). Following the introduction of whaling restrictions by the International Whaling Commission (IWC) in 1986, the species appears to have recovered in some parts of its range like in the eastern North Pacific (Monnahan, Branch, and Punt 2014), although it is still classified as endangered under the IUCN red list (Cooke 2018).

From a genetic perspective, it can be expected that the dramatic bottleneck reduced the genetic diversity within blue whale populations, increased the likelihood of inbreeding and altered gene flow between subpopulations and other species. However, high levels of genetic diversity have been revealed (Sremba et al. 2012), which was later also confirmed in genome‐wide heterozygosity analyses (Bukhman et al. 2024; Jossey et al. 2024; Wolf et al. 2022). In contrast, patterns of inbreeding measured by runs of homozygosity (ROH) indicated higher levels of inbreeding in the blue whale compared to other cetacean species, although these assessments were based on single genomes (Wolf et al. 2022, Bukhman et al. 2024).

This picture is complicated by evidence of gene flow between blue whales and fin whales, *Balaenoptera physalus* (Jossey et al. 2024; Pampoulie et al. 2021) and among different blue whale subspecies (Attard et al. 2012). In the latter case, neither the extent of gene flow nor its impact on the genetic diversity of certain blue whale subspecies is known. While genetic exchange between the Antarctic blue whale and the Western Australian pygmy blue whale have been studied in greater detail (Attard et al. 2012), there is no information about admixture between the Northern Hemisphere blue whale populations. Furthermore, conservation genomic analyses to assess genetic diversity, inbreeding and mutational load can provide valuable information on the genetic viability of a population and could help to channel further conservation efforts.

This study aims to estimate the genomic isolation between the Northern Hemisphere blue whale populations from the North Atlantic and North Pacific and compare the extent of genetic differentiation to the morphologically distinct and recognised Western Australian pygmy blue whale subspecies. Twenty blue whale specimens gathered from the North and equatorial Pacific (Pac) as well as Western Australian (WAus) pygmy blue whale populations were genome‐sequenced and analysed together with 12 published genomes of blue whales. This includes 11 individuals of the North Atlantic (Atl) populations (Jossey et al. 2024) and 1 additional from the North Pacific (Bukhman et al. 2024). The sampling was further complemented by three genomes of the closely related sei whale ( *Balaenoptera borealis* ), of which one is sequenced in this study and two were published by Árnason et al. (2018). This sampling allowed for characterising the extent of gene flow and hence admixture between the populations, determining the degree of genetic distance and estimating their phylogenetic relationships. Furthermore, the viability of these populations was studied by evaluating genome‐wide heterozygosity and by studying the scope of inbreeding based on ROH. These results enabled the first evaluation of the taxonomic status of the two Northern Hemisphere blue whale populations in the context of another recognised subspecies and in the context of the guidelines for subspecies delineation in Cetacea (Morin et al. 2023; Taylor et al. 2017). Through this process, we provide a comprehensive framework for population genomic studies to effectively guide conservation efforts. This is particularly important for endangered marine mammals or other threatened organisms that cannot be assessed by the means of classical observation.

## 2 Materials and Methods

### 2.1 Sampling, DNA, Library and Sequencing

**FIGURE 1:**
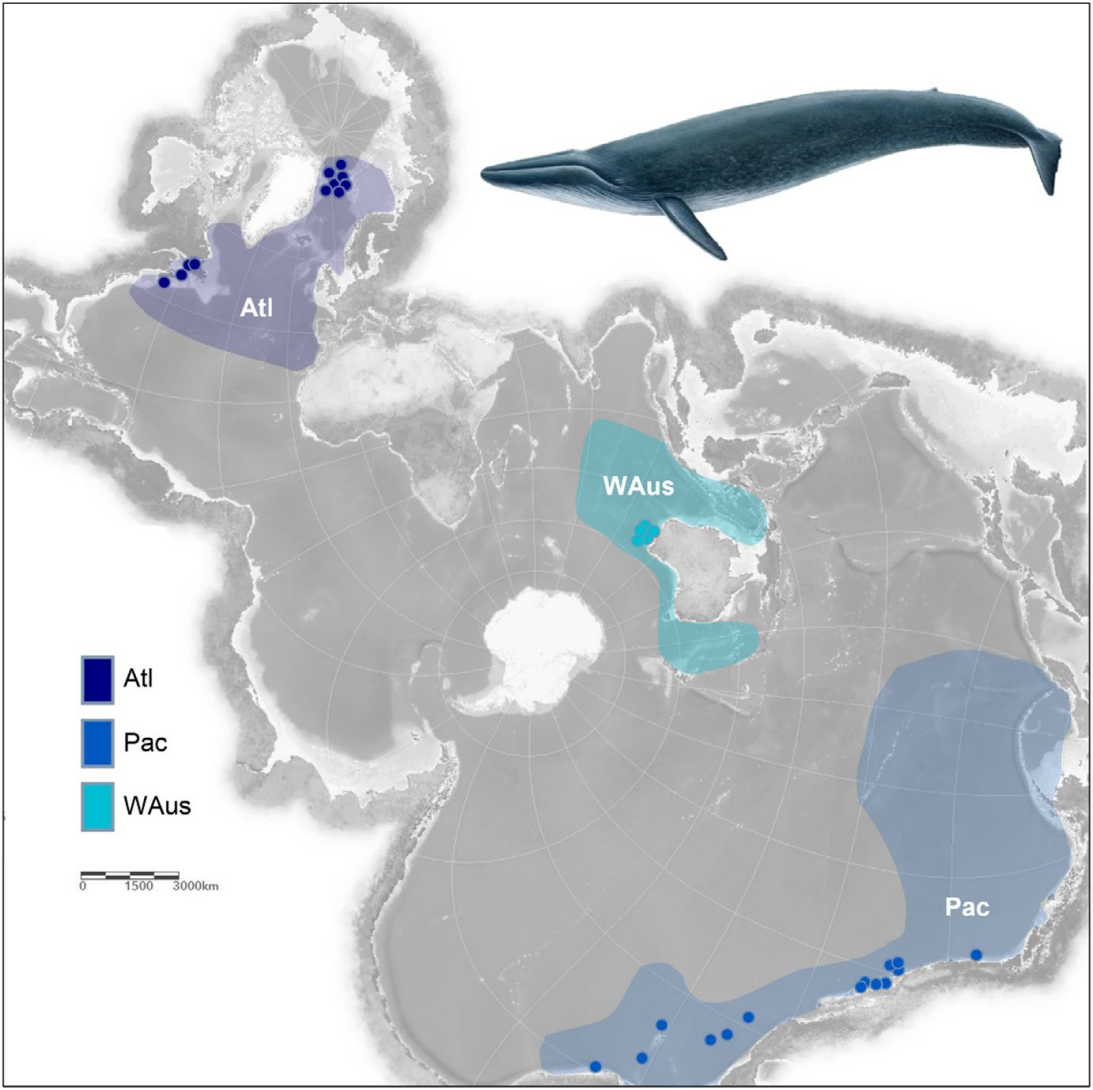
Geographical distribution of sampling locations of blue whale individuals analysed in this study. Colours were assigned to the respective oceanic region: Dark blue: North Atlantic (Atl); light blue: North and equatorial Pacific (Pac); cyan: Western Australia (WAus). Dots indicate the specific location of the sample, colour ranges represent the expected distribution of the population based on combined information found in: Atl: (McDonald, Mesnick, and Hildebrand 2006; Pike et al. 2013; Reeves et al. 2004; Silva et al. 2013); Pac: (Gilpatrick Jr. and Perryman 2008; McDonald, Mesnick, and Hildebrand 2006); WAus: (Branch et al. 2007; Leroy et al. 2021; McCauley et al. 2018; Sahri et al. 2022; Thums et al. 2022). Pacific and Western Australian samples were sequenced and provided in this study. North Atlantic samples were published in Jossey et al. (2024). Spilhaus projection map retrieved from ArcGis. Blue whale illustration made by Jón Baldur.

Twenty blue whale DNA samples were provided by the Marine Mammal and Sea Turtle Research (MMaSTR) tissue collection hosted at the NOAA Southwest Fisheries Science Center (SWFSC), USA. Corresponding metadata is provided in Figure 1; Figure S1 and Table S1. Given the cumulative nature of this collection, sampling procedures, sample handling and DNA extraction methods may vary. Nevertheless, all samples were stored at a minimum of −20°C and most DNA extractions were performed using a salting‐out protocol (Miller, Dykes, and Polesky 1988). All samples were collected as biopsies. North Pacific samples were collected between 1996 and 2006, always within the second half of the year (July–November). The more northern individuals of this population were usually sampled earlier in the year (July–September with one exception in November) while more southern individuals were sampled later in the year (October–November). Western Australian individuals were sampled early in the year (January–March) between 2000 and 2004.

The DNA from the single sei whale specimen was isolated from a primary tissue culture (passage seven) established by Úlfur Árnason from an Icelandic individual in 1980s. The fibroblast‐like cells were grown under standard conditions in rich Dulbecco's Modified Eagle's Medium (DMEM) supplemented with 5% fetal calf serum (FCS). After trypsinisation and resuspension in standard homogenisation buffer, the DNA was purified using a standard phenol/chloroform method (Sambrook and Russell 2006).

All Illumina paired‐end libraries were prepared by Novogene, Cambridge, United Kingdom using the nebnext dna library prep kit with a read length of 150 bp and an insert size of 350 bp. Illumina sequencing was performed on a NovaSeq 6000 platform targeting ~20x coverage per individual.

### 2.2 Genomic and Mitogenomic Variant Calling

The 20 generated short read datasets from Pacific blue whales, Western Australian blue whales and from the single sei whale individual were combined with publicly available short read datasets from 12 blue and 2 sei whales (Table S2, Árnason et al. 2018; Bukhman et al. 2024; Jossey et al. 2024). A comprehensive pipeline used to process the data and perform many of the here presented downstream analyses can be found on GitHub: mag‐wolf/RESEQ‐to‐Popanalyses/.

Short read data were trimmed for quality and adapter sequences using fastp v0.23.2 (Chen et al. 2018) with the options ‘‐g ‐3 ‐l 40 ‐y ‐c ‐p’. Trimmed reads were mapped to a repeat‐masked, high‐quality blue whale reference genome, constructed by the authors of the Vertebrate Genome Project (Bukhman et al. 2024). Mapping was performed using BWA MEM v0.7.17‐r1188 (http://bio‐bwa.sourceforge.net) and SAMtools v1.9 sort using default settings. Potential duplicates were removed, and read‐groups were added using the picard v2.21.2‐0 toolkit (https://broadinstitute.github.io/picard/). Mapping quality was inferred using Qualimap v.2.2.2 (Okonechnikov et al. 2016). Genotypes of genomes and mitogenomes were called in all individuals combined, and in each individual independently using bcftools v1.12 mpileup and bcftools v1.12 call with the respective ‘‐m’ or ‘‐c’ flag and minimum mapping‐ and base‐quality cut‐offs of 20 and 13, respectively. For the mitochondrial genome data, bcftools call was run with the ‘‐ploidy 1’ flag to account for haploidy. All inferred sites were filtered by excluding sites with divergent read coverage (> threefold and < 0.3‐fold of the expected individual mean coverage) and sites with more than 25% missing data using the bcftools filter function. The combined genotype set was then processed by removing multivariate and monomorphic sites to retrieve single nucleotide polymorphisms (SNPs) with vcftools v0.1.16 (Danecek et al. 2011) and by thinning SNPs to account for the effects of linkage disequilibrium using bcftools thinn function with a window size of 1000 bp. To evaluate variation of the mitochondrial control region, a commonly used marker for evaluation of subspecies within cetaceans, the respective 403 bp long area was extracted from the mitogenomic vcf file using bcftools view ‘‐r’ (Rosel, Dizon, and Heyning 1994). A comprehensive table of sequencing, mapping and variant calling statistics can be found in Table S3.

### 2.3 Population Structure and Gene Flow

Population genomic analyses were performed using the R‐based tool collection sambar v1.09 (de Jong et al. 2021). Using sambar's ‘findstructure()’ function, a principal coordinate analysis (PCoA) based on the ape‐5.3 package (Paradis and Schliep 2019) as well as an admixture analysis based on the LEA‐2.4.0 package (Frichot and François 2015) were conducted that also incorporated the elbow method on cross‐entropy scores to determine the optimal number of clusters (K, Figure S2). Gene flow was assessed by calculating the D‐statistic ‘ABBA‐BABA’ (Green et al. 2010) using a sliding‐window approach (https://github.com/ simonhmartin/genomics_general) to find local deviations from the assumed topology. The test was applied to the final SNP dataset and non‐overlapping sliding windows were set to a size of 100 kbp with a minimum of 100 SNPs. To exclude biases introduced by reference topology, all possible topologies were tested to avoid the chance of picking the wrong topology: ((Pac, WAus), Atl); ((Atl, WAus), Pac); ((Atl, Pac), WAus). A positive D value indicates an excess of asymmetric allele patterns (‘ABBA’ or ‘BABA’) that deviate from the expected pattern (‘AABB’), which typically groups two closely related populations separately from a third, more distantly related population and an outgroup.

Additionally, sambar's ‘inferdemography()’ function was used to calculate f2 statistics, denoting the mean squared difference between allele frequencies of two populations, and f3 statistics, testing for signals of admixture between two populations (Patterson et al. 2012, Table S4). The f2 statistic represents the sum of all branch lengths connecting two taxa, where these branch lengths are additive in the presence of multiple alternative pathways due to admixture. Thus, f2 functions as an intermediate measure between genetic differentiation and gene flow tests, as it depends on both factors. However, its limitation is that it scales quadratically with the number of admixture events, making it less suitable for cases with frequent admixture (Peter 2016). In a f3‐statistics, a negative value indicates that the allele frequencies of a population are intermediate between two other populations, which is a hallmark of admixture. The advantage of the f3‐statistic over the D‐statistic (ABBA‐BABA test) is that admixture signals can be reliably inferred without an established phylogeny. However, the f3‐score is not insensitive to population size fluctuations, and signals decay over time as a function of effective population size (N_e_, de Jong et al. 2023). Doing so, all possible combinations of the three blue whale populations were tested as for the ABBA‐BABA analysis.

### 2.4 Genetic Differentiation and Phylogeny

The standard genetic distance D_XY_, after Nei (1972) between individuals was estimated from the vcf file containing all blue and sei whales and both monomorphic and polymorphic sites using the ‘expected sequence dissimilarity’ metric (Xia et al. 2009). A Jukes–Cantor correction was applied to correct for multiple substitutions. Other population‐level genetic differentiation metrics, namely F_ST_ (Bhatia et al. 2013; Hudson, Slatkin, and Maddison 1992) and d_A_ (Nei 1987) were calculated using sambar's ‘hudsonfst()’ function. F_ST_ is calculated to measure the amount of genetic variation that is explained by population structure while Net nucleotide divergence (d_A_) is calculated to evaluate the amount of genetic divergence (D_XY_) corrected for within‐group genetic diversity. A percent diagnosability test (PD, Archer, Martien, and Taylor 2017), a measure of the ability of a random forest algorithm to correctly determine the group of a specimen based on molecular data, was done with mitogenomic sequences using the stratag v.2.4.905 package (Archer, Adams, and Schneiders 2017) in R. All genetic differentiation tests were done in a pairwise manner between all combinations possible: North Atlantic—North Pacific (Atl‐Pac), North Atlantic—Western Australia (Atl‐WAus) and North Pacific—Western Australia (Pac‐WAus).

Evolutionary trees were constructed with two different approaches. First, the D_XY_ values calculated above were used to construct a BIO‐neighbour‐joining (BIONJ) tree (Gascuel 1997) using the ape‐5.3 package (Paradis and Schliep 2019) and to generate a heatmap using ggplot2. Second, a dated phylogeny was constructed using whole mitogenomic sequences, retrieved by constructing consensus sequences from the previously called genetic variants of all blue and sei whales using the bcftools consensus function and the mitochondrial genome assembly of the blue whale (Bukhman et al. 2024, GenBank: CM018075.1) as reference. Mitochondrial data were specifically chosen for the dated phylogeny to investigate the more distant past of blue whale populations given the non‐recombining nature of mitochondrial DNA (de Jong et al. 2023). To add more calibration points, mitochondrial sequences for the fin whale ( *Balaenoptera physalus* , Wolf et al. 2022), humpback whale *Megaptera novaeangliae* , Tollis et al. 2019), grey whale ( *Eschrichtius robustus* , Árnason et al. 2018), minke whale ( *Balaenoptera acutorostrata* , Yim et al. 2014) and North Atlantic right whale ( *Eubalaena glacialis* ) were added by downloading respective read archives from NCBI (Table S5) and including them into the previously described pipeline for variant calling. BEAST2 v.2.7.6 (Bouckaert et al. 2014) was used to generate a dated phylogeny using a Bayesian MCMC approach, applying a gamma site model to account for site‐to‐site rate heterogeneity, a GTR substitution model and an optimised relaxed clock. Priors for each split in the outgroup were provided as normal distributed assuming a ‘Coalescent Constant Population’ tree prior. Divergence times for priors were collected from Árnason et al. (2018) and Wolf et al. (2023) assuming the topology presented in both studies. The blue whale divergence time was furthermore refined with the oldest known fossil record of a blue whale dated back to around 1.9 million years ago (Mya, Buchmann et al. 2017). BEAST2 was run with a 10 million‐generation long MCMC chain, sampling every 10th thousand tree. tracer v.1.7.2 (Rambaut et al. 2018) was used to check for chain stationarity and effective sample size (ESS) > 200 in all parameters. Sampled trees were merged into a maximum clade credibility tree using the treeannotator package distributed with BEAST2, and the tree was visualised in figtree v.1.4.4 (https://github.com/rambaut/figtree/). Eventually, the North Atlantic right whale was excluded to increase visibility of the rather short intra‐blue whale divergence times.

### 2.5 Genetic Diversity and Inbreeding

Genome‐wide heterozygosity was inferred from the individually called vcf files containing both monomorphic and polymorphic sites as the proportion of heterozygous sites per individual. An ANOVA test and a Tukey's post hoc test were performed to test if inter‐population differences were significant.

All other genetic diversity parameters such as nucleotide diversity, Tajima's D and Watterson's Θ were calculated on a window‐based approach using 100 kbp long, non‐overlapping sliding windows to account for region‐specific variants in the genome. Nucleotide diversity was calculated per population using the general_genomics tool collection (https://github.com/simonhmartin/genomics_general) using thresholds that exclude windows if they contain less than 100 SNPs in more than 50% of all individuals. Tajima's D neutrality test and the Watterson's Θ statistic were calculated using the ANGSD v0.931 tool collection following user recommendations (Korneliussen, Albrechtsen, and Nielsen 2014). Folded side frequency spectra (fSFS), necessary to calculate the number of segregation sites, were directly inferred from mapping files using a combination of the ‘‐dosaf 1’, ‘realsfs ‐fold 1’, ‘saf2theta’ and ‘thetastat’ functions. A more detailed description of these methods can be found in Korneliussen et al. (2013).

Runs of homozygosity were identified with darwindow (https://github.com/mennodejong1986/Darwindow), by using a sliding window approach to determine levels of heterozygosity per 20kbp window. Scaffolds smaller than 5Mbp were excluded from the analysis because they may be shorter than the expected ROH length. Validation of ROH‐calls was performed through visual examination (e.g., Super scaffold_9 depicted in Figure S3). ROHs shorter than 1 Mbp were not considered since they might also result from random processes other than inbreeding and since long ROH are more indicative of recent inbreeding events. The heterozygosity threshold was kept at the same level most of the time but was adjusted for four particularly heterozygous individuals (Pac: 5810; WAus: 23982, 23,984, 42,284; see Results). ROHs were sorted into 11 different length (Mbp) bins (1–1.5, 1.5–2, 2–2.5, 2.5–3, 3–3.5, 3.5–4, 4–5, 5–6, 6–8, 8–10 and 10‐open). Per bin, inbreeding coefficients (F_ROH_, after; McQuillan et al. 2012) were calculated as the proportion of the reference genome covered by the sum of all corresponding ROHs. A one‐way ANOVA test and a Tukey's post hoc test were used to detect significant differences between F_ROH_ bins of different populations, although tests were only possible up to bin number 7 (4–5Mbp), as later bins did not contain enough data to make meaningful comparisons.

## 3 Results

Whole‐genome short‐read datasets were generated for 14 northern and equatorial Pacific blue whale individuals and 6 individuals originating from Western Australian waters (Figure 1), as well as for 1 new sei whale individual from Icelandic waters. On average, 46 Gbp of data were sequenced per individual, equaling a 19‐fold coverage. This dataset was complemented by publicly available sequences of 11 North Atlantic blue whales (Jossey et al. 2024), another North Pacific individual (Bukhman et al. 2024) and 2 sei whale genomes (Árnason et al. 2018) resulting in a total set of 35 individuals represented by genome data (Tables S1 and S2). Mapping statistics for all individuals can be found in Table S3. From this, a set of 1.5 million high‐quality SNPs was called and used for many of the downstream analyses. In addition, mitogenomic sequences were extracted from all included individuals which contained 589 variable sites after filtering.

### 3.1 Population Structure

**FIGURE 2:**
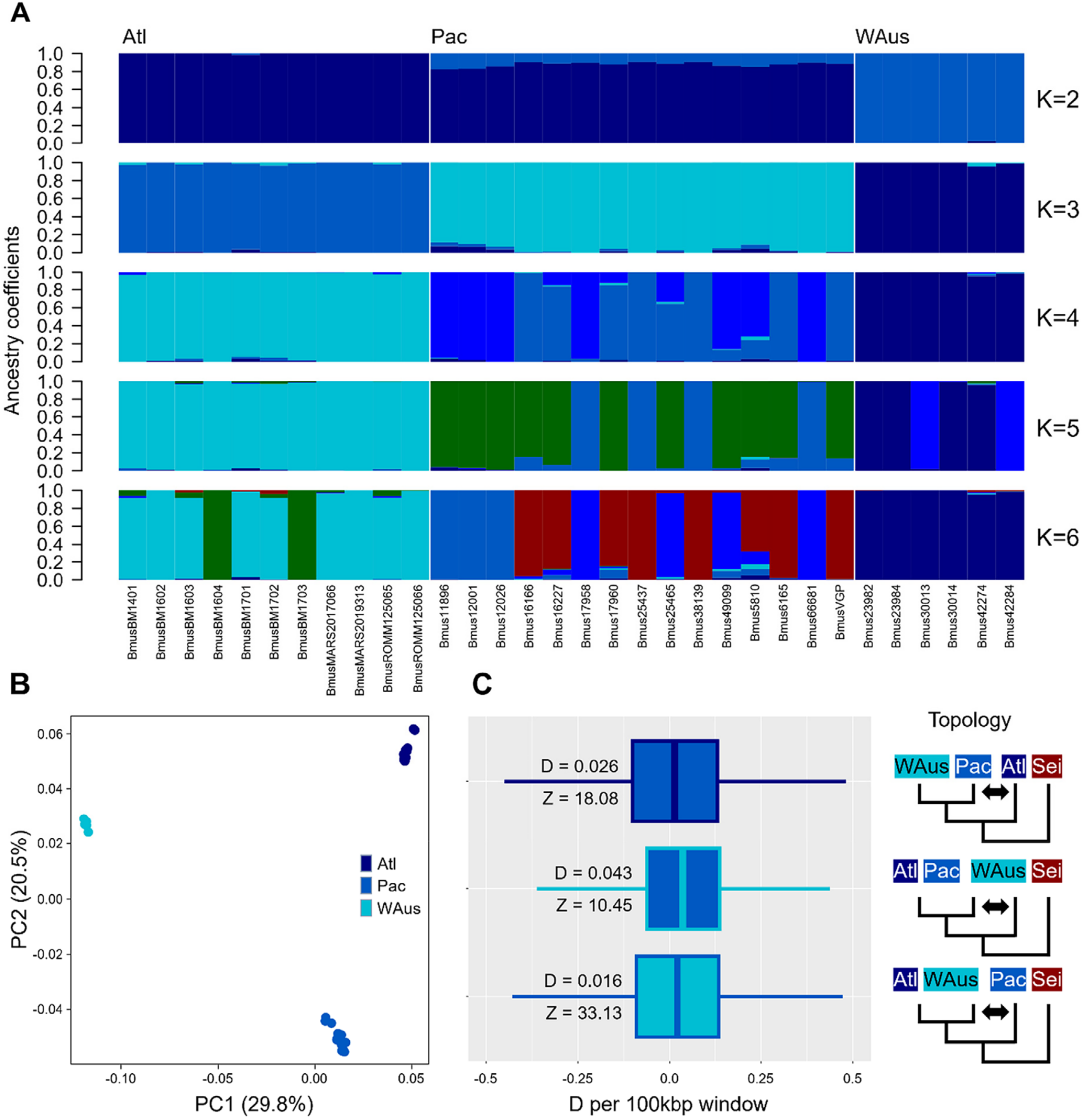
Population structure and gene flow analyses in blue whales from North Atlantic, Pacific and Western Australian waters. (A) An admixture‐like test generated with the LEA R package to infer ancestry coefficients assuming 2–6 ancestral populations. Apart from the clear separation into the three oceanic regions, no consistent pattern was found. Colours do not necessarily correspond to colours assigned to sample origin. (B) Principal Coordinates Analysis (PCoA) generated with the ape R package. All three oceanic regions were separated into distinct clusters. (C) D‐statistical assessment of gene flow between the three blue whale populations assuming the respective topology depicted on the right‐hand side. Results reflect the spectrum of values retrieved from 100 kbp window‐based analysis. All tests resulted in low but positive D values and high Z scores.

Estimates of ancestry coefficients and population structure were inferred using the R package LEA assuming 2–6 ancestral populations (K, Figures 2A and S2). Minimum cross‐entropy scores increased with K, and it was not possible to unambiguously identify a best‐fitting model using the elbow method. When assuming two populations (K = 2), a clear separation between the Western Australian pygmy blue whales and the two other sampled Northern Hemisphere oceans is evident. At *K* = 3, the two northern populations are further differentiated into an Atlantic and a Pacific population, consistent with the next‐lowest cross‐entropy score. A higher number of ancestral populations did not result in any meaningful clusters. A principal coordinate analysis (PCoA, Figure 2B) confirmed the existence of three distinct blue whale populations with the first two axes of differentiation. PC1 explained 29.8% of the variation and separated the Western Australian blue whales from northern blue whales while PC2 explained 20.5% of the variation and separated Atlantic from Pacific individuals.

### 3.2 Genetic Exchange

Signs of admixture were limited in the admixture analysis and mostly non‐consistent over different numbers of assumed populations (K, Figure 2A). In the *K* = 2 inference, all Pacific individuals showed minimal signs of admixture with the Western Australian blue whale, and these signs remain, albeit to a lesser extent, only in four individuals over higher values of K. Apart from this, no patterns over different values of K could be identified that would suggest clear signals of shared ancestry.

Gene flow analyses using a sliding‐window based D‐statistic ‘ABBA‐BABA’ approach were conducted over all three possible inferred topology‐combinations, using the sei whale as an outgroup (Figure 2C). The test resulted in slightly positive D values and Z‐scores over 3, indicating robust, albeit limited admixture. These results were consistent across all tested combinations: ((WAus, Pac), Atl) = 0.026; ((Atl, WAus), Pac) = 0.016; ((Atl, Pac), WAus) = 0.043. A calculation of f3‐statistics resulted in positive f3 values with high Z scores and significant *p*‐values regardless of the tested combination, indicating no detectable signals in any of the populations that would support an intermediate origin from the other two populations (Table S4B).

### 3.3 Genetic Distance and Phylogeny

**FIGURE 3:**
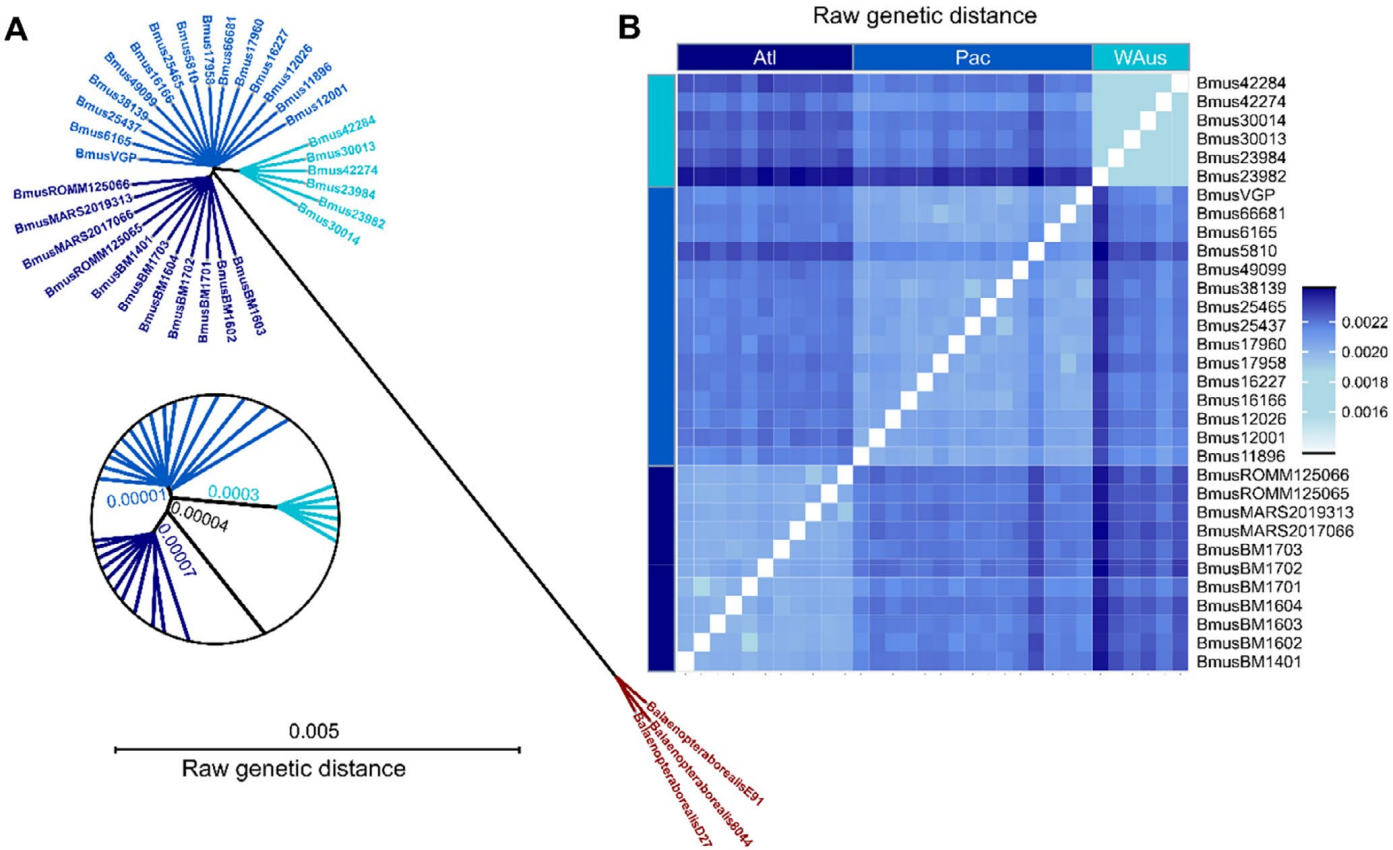
Genetic distances among blue whales from different oceanic regions, including a sei whale outgroup. (A) BIONJ‐based phylogeny constructed with the ape R package. All oceanic regions form distinct clusters and branch lengths indicate the highest amount of genetic distance between Western Australian blue whales and other populations. (B) Heatmap representing pairwise genetic distances between blue whale individuals. The largest distance was found between North Atlantic and Western Australian individuals. Western Australian individuals displayed a comparable low population‐internal genetic distance. Two outlier individuals were found with a higher genetic distance to all other individuals, namely 5810 (Pac) and 23,982 (WAus).

Pairwise genetic distances were calculated based on the total set of variances including monomorphic sites. The phylogenetic tree based on BIONJ clustering resolved all three blue whale populations as in the population structure analyses and showed a genetic distance of about 0.8% between the sei whale and the inferred blue whale populations (Figure 3A). Distances between populations (here indicated as branch lengths) were small compared to the species level distance, while Western Australian pygmy blue whale individuals showing the highest level of population‐specific signals compared to the two northern populations.

A heatmap generated using the pairwise genetic distances depicts a similar situation (Figure 3B). More specifically, the distance between the North Atlantic blue whale and the Western Australian pygmy whale population was the highest (Atl‐WAus: ~0.024%), followed by North Pacific and Western Australia (Pac‐WAus: ~0.023%) and formally capped off by the lowest distance between North Atlantic and North Pacific (Atl‐Pac: ~0.021%). Interpopulation distances were lower in general and lowest between Western Australian individuals, possibly reflecting the less distributed sampling of the population. Furthermore, two outlier individuals were found with higher levels of individual‐specific genetic signals, namely individual number 5810 (Pac) and 23,982 (WAus), which also appear distinctly in other analyses and may reflect gene flow to populations for which no samples could be obtained.

**FIGURE 4:**
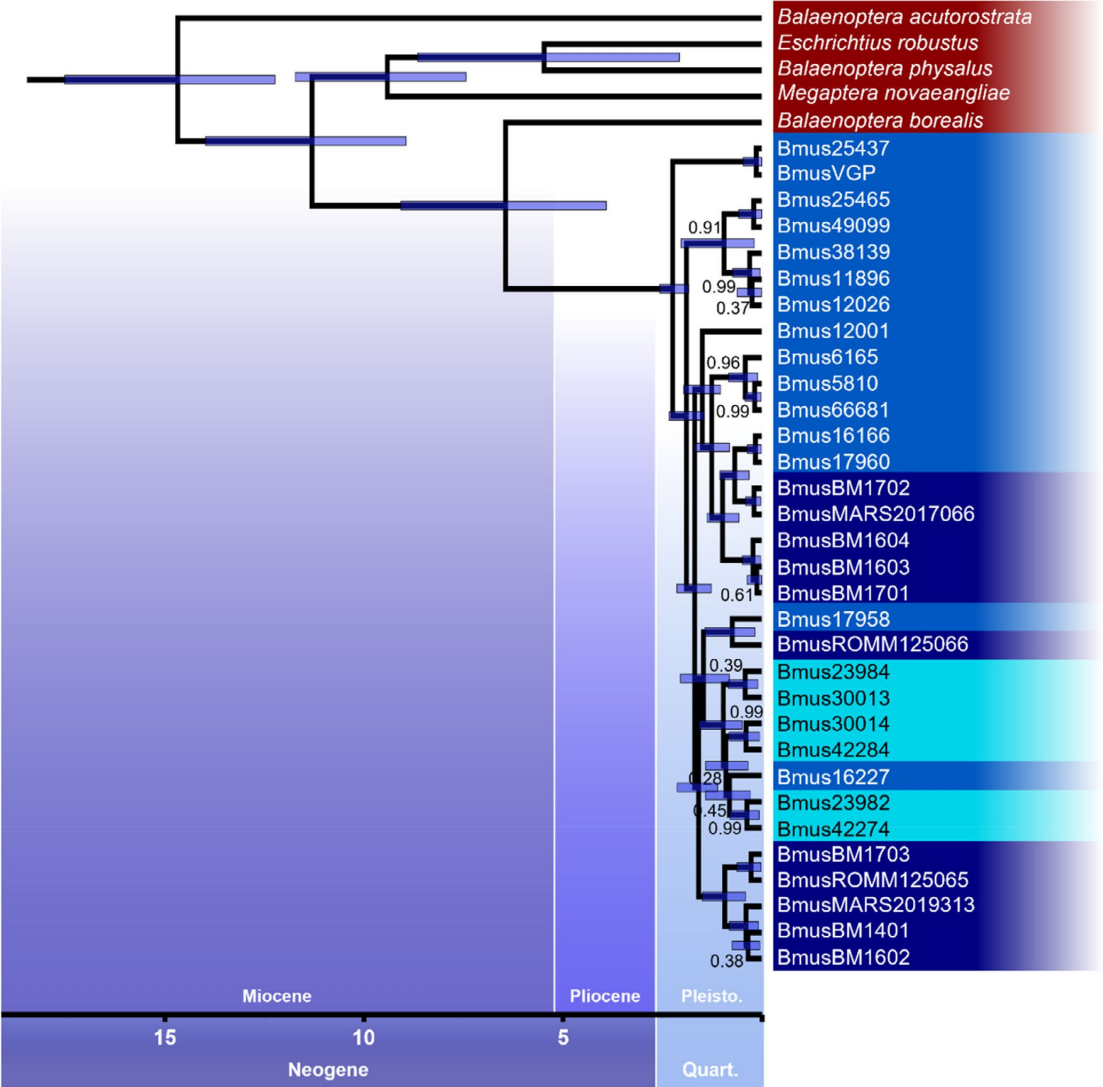
Dated phylogeny based on mitogenomic data using a Bayesian MCMC approach. Tree created with BEAST2 using other rorquals and baleen whales as outgroup. Divergence time priors for the outgroup were compiled from fossil records (Buchmann et al. 2017) and other molecular‐clock divergence time estimates found in Árnason et al. (2018), Buchmann et al. (2017), and Wolf et al. (2023). Bayesian posterior probabilities (BPP) for each node are shown if BPP ≠ 1. Individual background colours represent sampling location: Dark blue: Atl; light blue: Pac; cyan: WAus; red: Other species. Divergence times for blue whales were estimated to be between 100 thousand and 2.2 million years ago, roughly in line with the oldest known blue whale fossil (Buchmann et al. 2017, ~1.9 Mya). Overall, none of the tested populations were resolved as monophyletic. Two North Pacific individuals were found in North‐Atlantic and Western Australian clades, while the North Atlantic population was found to be polyphyletic, separated into two distinct groups which group together with the two other populations, respectively. Given the nature of mitochondrial sequences as non‐recombining and maternally inherited, the tree likely depicts older dispersal events that followed glacial periods causing uninhabitable polar regions (e.g., see Morin et al. 2018; de Jong et al. 2023).

The dated phylogeny, based on a Bayesian MCMC approach using mitogenomic sequences and a larger collection of outgroup species, resulted in no monophyletic, population‐specific clusters compared to the genetic distance BIONJ tree constructed with whole‐genome data (Figure 4). Providing calibration points for every outgroup split, the divergence of blue whales was dated to be at roughly 2.23 Mya with a 95% HPD (Highest Posterior Density) interval of 1.8–2.5 Mya, roughly around the provided fossil calibration point of ~1.9 Mya (Buchmann et al. 2017) and the beginning of the Pleistocene. The first individuals found to diverge were whales from the North Pacific, followed by a larger cluster of North Atlantic and North Pacific whales. A third major group contains another larger group of North Atlantic whales as well as the Western Australian population. Two North Pacific individuals were sister to clades containing representatives of the other two populations. Low Bayesian posterior probabilities (BPP) were found mainly around and within the Western Australian population, all other nodes had a BPP = 1.

### 3.4 Genetic Differentiation

Genomic differentiation estimates using F_ST_ statistics (Bhatia et al. 2013; Hudson, Slatkin, and Maddison 1992), net nucleotide divergence d_A_ (Nei 1987, Table 1) and f2 statistics (Patterson et al. 2012, Table S4A), suggested a high fixation rate but low divergence between all blue whale populations. F_ST_ values, calculated to measure the amount of genetic variation explained by population structure between the different blue whale populations, ranged from 0.06 to 0.15, with Atlantic and Pacific having the lowest (Atl‐Pac: 0.062), Pacific and Western Australia having in between (Pac‐WAus: 0.125) and Atlantic and Western Australia having the highest (Atl‐WAus: 0.147) genetic differentiation. Net nucleotide divergence (d_A_), a measure of genetic divergence corrected for within‐group genetic diversity, was again lowest in the comparison of the two northern populations (Atl‐Pac: 0.014%), and higher in comparisons with the Western Australian blue whales (Atl‐WAus: 0.039%; Pac‐WAus: 0.032%). The f2‐statistics, denoting the mean squared difference between allele frequencies of two populations, yielded the lowest values between the Atlantic and Pacific population (Atl‐Pac: 0.02) and higher values were noted between each of both northern populations and the Western Australian pygmy blue whale population (Atl‐WAus: 0.051; Pac‐WAus: 0.043).

**TABLE 1:** Differentiation estimates inferred for 35 whole genome re‐sequencing datasets, including mitogenomic and control region data from 32 blue whales of three different oceanic regions (North Atlantic, Pacific and Western Australia), as well as three sei whale samples. The table includes pairwise comparisons of d_A_ (Nei 1987) values, mean F_ST_ (Bhatia et al. 2013; Hudson, Slatkin, and Maddison 1992) and PD (%, Archer, Martien, and Taylor 2017). Following Taylor et al. (2017) and Morin et al. (2023), the lower threshold of dA for subspecies definition, > 0.004 (control region) and > 0.0006 (mitogenomic), was met only once in the control region data between North Atlantic and Western Australian blue whales. The lower threshold for the mitogenomic PD test of > 80% (following Morin et al. (2024), assuming a reduced sample size) was met in all pairwise tests but was highest between both northern populations.

Data	Population	Pac	WAus	Sei	Stat.
Genomic	Atl	0.0001	0.0004	0.008	d_A_
	Pac	—	0.0003	0.008	
	WAus	—	—	0.0082	
Mitogenomic	Atl	0.0002	0.0004	0.0318	
	Pac	—	0.0002	0.0319	
	WAus	—	—	0.0323	
Ctrl. region	Atl	0.0009	0.0043	0.0338	
	Pac	—	0.0024	0.0394	
	WAus	—	—	0.0449	
Genomic	Atl	0.0619	0.1470	—	F_ST_
	Pac	—	0.1247	—	
Mitogenomic	Atl	0.11	0.227	—	
	Pac	—	0.145	—	
Ctrl. region	Atl	0.0709	0.3129	—	
	Pac	—	0.2648	—	
Mitogenomic	Atl	96	94	—	PD (%)
	Pac	—	86	—	

Differentiation statistics of the mitochondrial genomes were generally consistent with their genome‐wide equivalents but showed a lower divergence between Pacific and Western Australian blue whales, lower than between Atlantic and Pacific (Table 1). The F_ST_ value of the Atlantic and Pacific pair had the lowest fixation rate (Atl‐Pac: 0.11), followed by the pair of Pacific and Western Australian blue whales (Pac‐WAus: 0.145) and capped off by the Atlantic and Western Australian pair (Atl‐WAus: 0.227). Net nucleotide divergence, d_A_ was lowest between the Pacific and Western Australian individuals (Pac‐WAus: 0.0151%). Atlantic and Pacific individuals featured a d_A_ of 0.0196% (Atl‐Pac) and Atlantic and Western Australian individuals diverged by 0.0362% (Atl‐WAus). Conversely, percent diagnosability (PD, Archer, Martien, and Taylor 2017), was found to be highest between the two northern populations (Atl‐Pac: 96%), intermediate between North Atlantic and Western Australia (Atl‐WAus: 94%) and lowest between North Pacific and Western Australian blue whales (Pac‐WAus: 84%).

As previous comparisons were often based on the mitochondrial control region sequence (Rosel, Dizon, and Heyning 1994), the specific region was extracted from the mitogenomic set of variation and the analysis was rerun with similar results. F_ST_ values were again lowest for the Atlantic and Pacific pair (Atl‐Pac: 0.071), intermediate for the Pacific and Western Australian pair (Pac‐WAus: 0.265) and the Atlantic and Western Australia pair had the highest fixation rate (Atl‐WAus: 0.313). Similar to the whole genome results, Atlantic and Pacific featured the least genome‐wide divergence with d_A_ = 0.086% (Atl‐Pac), while Atlantic and Western Australian blue whales were most diverged with d_A_ = 0.431% (Atl‐WAus). Like for the whole genome results and contrary to the whole mitogenome values, the pair of Pacific and Western Australia showed intermediate divergence with d_A_ = 0.24% (Pac‐WAus).

### 3.5 Genomic Diversity and Inbreeding

**FIGURE 5:**
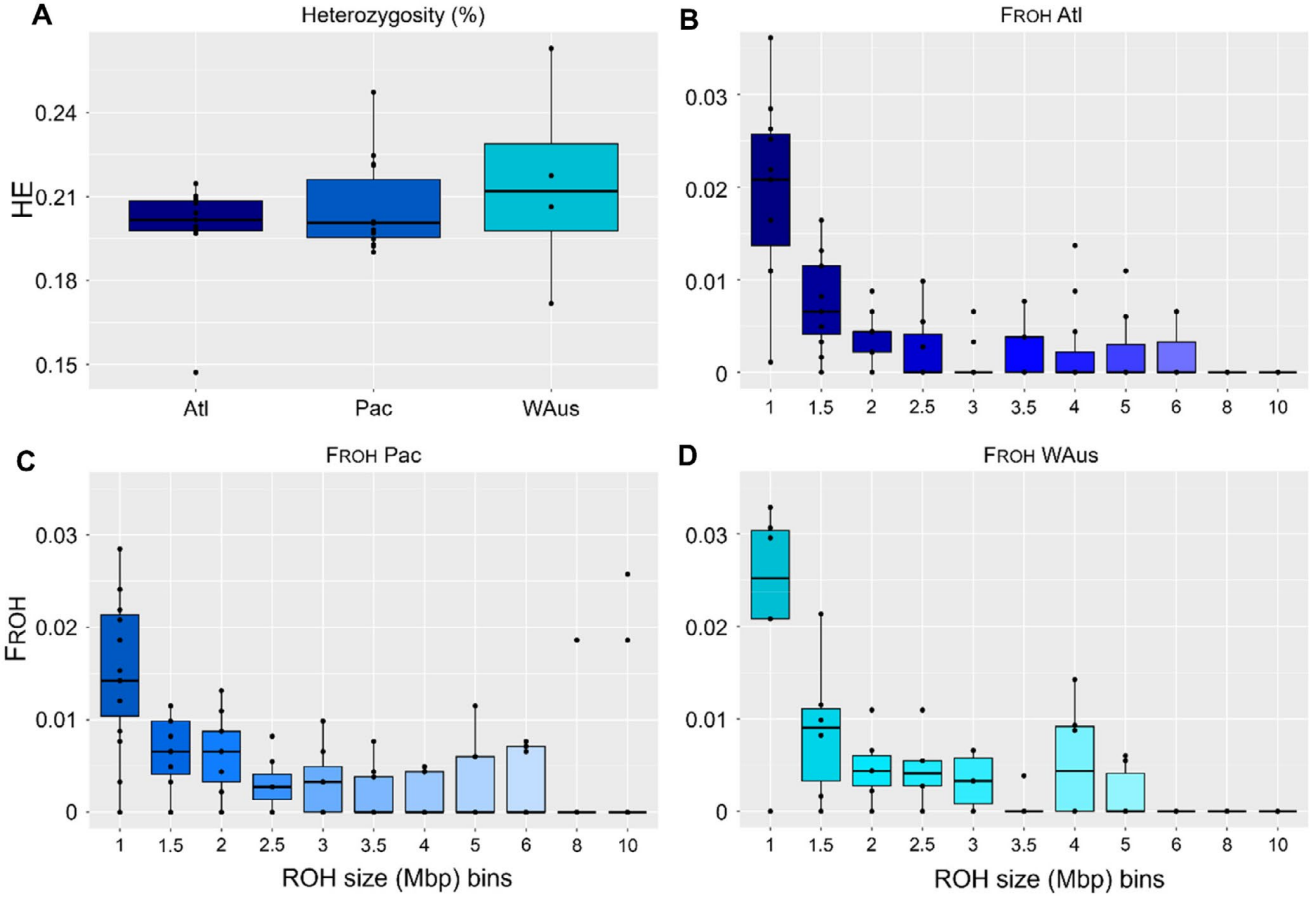
Genomic diversity and inbreeding coefficients in the three inferred blue whale populations from different oceanic regions (North Atlantic, Pacific and Western Australia). (A) Genome‐wide levels of heterozygosity were similar in Pacific and Atlantic individuals (Pac = 0.206, Atl = 0.199) and highest and most diverse in Western Australian individuals (WAus = 0.215), although differences were non‐significant. (B–D) Inbreeding coefficients based on runs of homozygosity (F_ROH_) sorted in different size‐bins (1–10 Mbp) compared over the three inferred blue whale populations. All populations featured ROHs in bins ranging from > 1 to < 6 Mbp. ROHs over 8 Mbp were only found in the Pacific cohort. In bins that allow for statistical comparisons, no significant differences between whale populations were found.

Genomic diversity within the populations was assessed by the means of genome‐wide heterozygosity, nucleotide diversity (π), Tajima's D, Watterson's Θ and inbreeding coefficients based on runs of homozygosity (F_ROH_, Figure 5, Table 2). Heterozygosity was comparable between the two northern populations with a mean value of 0.20% and 0.21% in the Atlantic and Pacific population, respectively (Figure 5A). Western Australian pygmy blue whales showed the highest variance and the highest mean value of 0.22% but were not significantly higher compared to the other populations (ANOVA: *f* = 0.94, *p* = 0.4; Tukey: *p* = 0.4). Three individuals were excluded due to their exceptionally high level of heterozygosity that may have been caused by hybridisation with a non‐sampled population, or another species rather than representing the natural heterozygosity of the respective population: Pacific: 5810; Western Australian: 23982 and 42,284. These high levels of heterozygosity cannot be attributed to differences in data quality, as these individuals fall within the range of other individuals in terms of sequencing and mapping quality (Table S3). Nucleotide diversity, defined as the mean pairwise difference within individuals of a population, was found to closely resemble the overall heterozygosity levels, with the Pacific and Western Australian populations having slightly lower values compared to mean heterozygosity (Pac = 0.20%, WAus = 0.21%).

**TABLE 2:** Diversity statistics inferred for the three blue whale populations North Atlantic, Pacific and Western Australia. The table includes statistics for mean genome‐wide heterozygosity in % (He), nucleotide diversity in % (π), Tajima's D, Wattersons Θ and inbreeding coefficients based on ROH of 1 Mbp or longer.

Metrics	Population		
	Atl	Pac	Waus
Mean He	0.199	0.206	0.215
Nuc. div (π)	0.199	0.204	0.212
Tajima's D	0.882	0.594	0.857
Watterson Θ	121.1	121.3	122.4
F_ROH_ (> 1 Mbp)	0.043	0.052	0.048

Neutrality tests, meant to distinguish whether a population is evolving neutrally or not, were based on the folded side frequency spectrum (fSFS, Korneliussen et al. 2013) to estimate the number of segregating sites. This test revealed a similar positive Tajima's D for the Atlantic and Western Australian population (Atl = 0.88, WAus = 0.86) and a lower but still positive value for the Pacific population (Pac = 0.59), indicating an excess of variation in the observed diversity compared to the expected diversity in all inferred groups. Watterson's Θ, a measure of the expected nucleotide diversity within a group or population, was similar in all three populations, ranging from 121.1 (Atl), 121.3 (Pac) and 122.4 (WAus).

Inbreeding coefficients were determined using a heterozygosity threshold of 0.06 that provided the best fit for most individuals. Four individuals were particularly heterozygous (Pac: 5810; WAus: 23982, 23,984, 42,284) and respective thresholds were changed to a best fit at 0.12. Overall, there were fewer ROHs as ROH size increased and no prominent nor significant differences between the blue whale populations (Figure 5B–D). The Pacific individuals featured the lowest amount of short ROHs (< 2 Mbp) and the overall longest ROHs with a record of a 17 Mbp long ROH in the individual 17,960 (Pac), suggesting influences of more recent inbreeding. By contrast, the Western Australian pygmy blue whales had the highest number of short ROHs and did not feature ROHs longer than 5.5 Mbp.

## 4 Discussion

This study compared the genomes of North Atlantic and North Pacific blue whales with those of the Western Australian pygmy blue whale subspecies and found not only an overall high degree of isolation as indicated by low amounts of gene flow and high F_ST_ values, but also a lack of genomic and mitogenomic divergence by the means of d_A_. Given the context provided here with the pygmy blue whale as well as other distinct subspecies (Morin et al. 2023), a proposal is made that the two northern populations represent nearly equally independent branches of the overall blue whale distribution.

### 4.1 Strong Isolation Separates Atlantic and Pacific Blue Whales

The genomic separation among all three populations coincides with their geographical distance. A connection between the North Atlantic and Pacific blue whale populations has not been reported and seems unlikely given the Artic sea ice coverage and the blue whales' dependency on high ocean productivity in areas connected to deep sea ocean dynamics (Branch et al. 2007). This suggests a long geographical distance between the Atlantic and Pacific populations via the Southern Hemisphere, comparable to, or even larger than the distance towards the Western Australian pygmy blue whales. Evidence of genetic exchange was limited among all three groups with a slightly higher connectivity between the North Pacific population and the Western Australian pygmy blue whale population when compared to the North Atlantic population. Given the lack of ocean productivity in the waters of the central Pacific, the lack of songs recorded in the central Pacific and the lack of recorded latitudinal migrations, it can be assumed that the most likely route for genetic exchange between all three populations would be via hybridisation zones with other populations (Bailey et al. 2009; Branch et al. 2007; Hucke‐Gaete et al. 2018; McDonald, Mesnick, and Hildebrand 2006). This could, for example, happen in a stepwise manner and over multiple generations of interbreeding with the Antarctic blue whale subspecies (*B. m. intermedia*). This is at least partially supported by reports of a hybridisation zone between the Western Australian pygmy blue whales and the Antarctic subspecies (Attard et al. 2012) as well as the occurrence of Antarctic blue whales in the south East Pacific (LeDuc et al. 2017). As the literature does not report a hybridisation zone between North Atlantic and Southern Hemisphere populations, likely due to the absence of a major population in the equatorial Atlantic (Branch et al. 2007), one interpretation is that the increased signals of gene flow between North Pacific and Western Australia populations may result from their more frequent interaction with the Antarctic subspecies.

### 4.2 Low Genetic Differentiation Among all Populations

Genomic F_ST_ values between the three blue whale populations not only fall within the range of other recognised cetacean subspecies (0.004–0.012, Leslie and Morin 2018; 0.18–0.24, Lah et al. 2016), but also into the range of proposed populations (0.09, Foote et al. 2016; 0.018–0.197 Onoufriou et al. 2022). The same is found for mitogenomic and mitochondrial control region F_ST_ values compared to other subspecies (Leslie and Morin 2018) and populations (Onoufriou et al. 2022). These F_ST_ values were also higher compared to values between accepted fin whale subspecies (0.0046–0.0184, Archer et al. 2019), demonstrating F_ST_'s unreliability for subspecies definition in cetaceans (Rosel et al. 2017). For mitochondrial control region F_ST_ values, direct comparisons between other blue whale subspecies exist that fit the here observed range (0.06–0.18; LeDuc et al. 2007; Sremba et al. 2012; Torres‐Florez et al. 2014).

The genomic and mitogenomic net nucleotide divergence; however, not only showed a lower divergence between the Northern Hemisphere populations but were also drastically lower when compared to other comparable cetacean data, like a d_A_ of 0.0097 for Cuvier's beaked whales from different ocean basins (Onoufriou et al. 2022) and a d_A_ of 0.0036–0.0072 for fin whales from different ocean basins (Archer et al. 2019). These values also fall below the recently proposed lower boundary for subspecies delimitation using mitogenomic data (d_A_ > 0.0006, Morin et al. 2023). Nevertheless, when compared to other recognised subspecies of blue whale also reported in Morin et al. (2023), it is evident that blue whale subspecies generally fall below this threshold, with the values presented here falling within the range observed for all other subspecies (Figure S4).

The low genetic differentiation observed uniformly across all tested blue whale populations suggests that the underlying causes for this lack of variation may be species‐wide in nature. The main factors determining the rate of divergence are population size, inter‐ and intrapopulation admixture, generation times and substitution rates (Edelman and Mallet 2021; Lanfear, Kokko, and Eyre‐Walker 2014). Contrary to today's situation, pre‐Anthropocene populations may have been rather large as indicated by demographic models (Árnason et al. 2018; Bukhman et al. 2024; Jossey et al. 2024) and by their high genetic diversity which is also indicative for larger populations in their demographic past (Árnason et al. 2018; Teixeira and Huber 2021; Wolf et al. 2022). This relationship between demographic past and genetic differences would also explain the slightly increased levels of differentiation found in the Western Australian pygmy blue whale because their population sizes may have been lower throughout their history (Attard et al. 2015). The highly migratory nature (Hucke‐Gaete et al. 2018) of blue whales, their longevity of over 90 years (Sears and Perrin 2009) and their low reproductive rates (Taylor et al. 2007) could further reduce divergence.

The percent diagnosability (PD) test, contradicts this pattern, indicating a high level of diagnosability between all populations, especially between the two northern populations, with the lowest found between the North Pacific and Western Australian group. These values are all above the proposed threshold of 80% at lower sample sizes and are similar or even higher than other subspecies of blue whales, especially compared to tests involving the Antarctic subspecies (Morin et al. 2023). The diagnosability between the northern populations even meets the stricter threshold of PD > 95% when assuming larger sample sizes which may reflect the larger sample sizes of both northern populations in this study.

### 4.3 Phylogenetic Analyses Reveal a More Admixed Past

The time‐calibrated phylogeny based on mitogenomes revealed more distant episodes of maternally mediated gene flow, as this data represents a single, non‐recombining locus which is maternally inherited (de Jong et al. 2023). The overall divergence of blue whales was estimated to be at around 2.23 Mya, which roughly coincides with the beginning of the Pleistocene (2.58 Mya). The early Pleistocene is marked by the closure of the Isthmus of Panama (Coates et al. 1992) and the onset of re‐occurring glacial periods (Paillard 2001), both massively changing oceanic currents and likely exerted a selective pressure on baleen whales (Ludt and Rocha 2015). This selection may have favoured long‐distance migratory behaviour and body gigantism (Slater, Goldbogen, and Pyenson 2017). Considering these events, the lack of population‐specific clusters in the phylogenetic tree could be explained by an increased gene flow which may have been prevalent during periods of glacial maxima, when Arctic and Antarctic habitats became uninhabitable for the more polar populations. These glacial cycles would also result in multiple re‐colonialisation events of polar oceanic regions, potentially explaining the more ancient splits within the northern populations (see also Morin et al. 2018). Nevertheless, these admixture events likely occurred in the more distant past, as the gene flow analyses only showed little genetic exchange between all populations, and as the BIONJ tree based on genome‐wide distances resolved the populations as monophyletic. Therefore, it can be assumed that this analysis does not oppose a current strong isolation and a potential subspeciation.

### 4.4 Two Potential Northern Hemisphere Subspecies

In a special issue of Marine Mammal Science (Volume33, Issue S1), Taylor and colleagues proposed guidelines for taxonomic decisions for cetacean species that ensure consistency in the use of molecular data (Taylor et al. 2017). In doing so, Taylor and colleagues proposed thresholds for two statistics (d_A_ and PD) using mitochondrial control region data (Taylor et al. 2017), which were later updated with mitogenomic data (Morin et al. 2023).

In cases of a recent split or large population size, these guidelines still recommend subspecies separation, even if one of both thresholds is not met, provided there is supporting evidence from other sources. Apart from the possibility of a large historical population size as detailed above, a more recent split, for example, after the Last Glacial Maximum (LGM), seems plausible too given the ancient admixture events found in the mitogenomic phylogeny. This scenario is further supported by other demographic assessments of the Western Australian pygmy blue whale and the Antarctic blue whale, which estimated their split at around 20,000 years ago (Attard et al. 2015).

Morphological differences between the two Northern Hemisphere populations are not known as no direct comparison between both groups has been attempted thus far. The reasons for this could be logistical challenges posed by their enormous body sizes and the general inaccessibility of pelagic animals (Taylor et al. 2017). Other lines of evidence for subspecies differentiation are acoustic patterns that are distinct between both northern oceans (McDonald, Mesnick, and Hildebrand 2006; Mellinger and Clark 2003). Songs of North Atlantic blue whales consist of tonal, single‐phrased, low‐frequency calls within the 15–20 Hz range while North Pacific songs contain multiple and often also pulsed phrases (20–120 Hz) that can differ between different localities in the North Pacific (McDonald, Mesnick, and Hildebrand 2006; Mellinger and Clark 2003; Stafford, Nieukirk, and Fox 2001).

Overall, the genetic isolation and diagnosability between the two Northern Hemisphere blue whale populations are comparable to, or even exceed those of the recognised Western Australian pygmy blue whale. Although genetic differentiation between the northern populations was lower, they were still comparable to the differences found in tests including the Western Australian outgroup and align with those observed in other blue whale subspecies (Figure S4, Morin et al. 2023). Therefore, a decision must be made to either revise the subspecies status of the pygmy blue whale based on the low genomic differentiation presented here, or to designate new subspecies. Given that these levels of genetic divergence have resulted in morphologically distinct traits in the pygmy blue whale, alongside reported acoustic characteristics distinguishing both populations, we would support the latter option. Finally, recognition of additional subspecies may also enhance conservation efforts, as previously outlined (Taylor et al. 2017).

In accordance with the International Code of Zoological Nomenclature (ICZN) priority principle, the name for the North Atlantic subspecies should be left as *Balaenoptera musculus musculus* due to the first scientific name of the blue whale formulated by Carl von Linné (1758). Apart from the diagnosable molecular patterns, characteristic features for the Atlantic blue whale (*B. m. musculus*) would be its song pattern as outlined in McDonald, Mesnick, and Hildebrand (2006) and Mellinger and Clark (2003). Type specimens of the North Atlantic blue whale are preserved in the Natural History Museum, London, Gray 1857, Catalogue No.: NHMUK ZOO 1935.6.24.1. and Flower 1865, Catalogue No.: NHMUK ZOO ZD 1865.8.23.1.

A North Pacific blue whale specimen was first described as *Sibbaldius sulfureus* by Scammon and Cope (1869), although this description has been generally taken as synonymous to *Balaenoptera musculus* (Ruud 1956). The genus name ‘*Sibbaldius*’ references its first description by Robert Sibbald in 1692 (Ruud 1956) and the species name ‘*sulfureus*’ derived from the yellowish (‘sulfur’) coat of diatoms on its underside. These diatoms seemed to be especially frequent in the colder waters of the North Pacific and Antarctica (Bennett 1920; Omura 1950; Scammon and Cope 1869) and resulted in the common name ‘Sulphurbottom’ used by whalers in the respective oceanic regions. Therefore, we propose *Balaenoptera musculus sulfureus* Cope 1869 as the name for the North Pacific blue whale subspecies. Characteristic song patterns include one of two characteristic song patterns specific for individuals of the North Pacific (McDonald, Mesnick, and Hildebrand 2006; Stafford, Nieukirk, and Fox 2001). A type specimen was not collected in the original description by Cope, and the designation of a new holotype would be required before a formal description can be made.

In the case of the North Pacific blue whale (*B. m. sulfureus*), it should be emphasised that it is currently not possible to differentiate between blue whales from the north and south East Pacific at the coast of Chile. The sampling in this study spans over multiple seasons and includes specimens collected in the Eastern Tropical Pacific. It is therefore possible, that the current analyses include individuals of both populations as they share this common breeding ground (Bailey et al. 2009; Hucke‐Gaete et al. 2018). Until comparable data from South Pacific individuals becomes available, it is possible that yet another subspecies may exists (LeDuc et al. 2007, 2017). Nevertheless, even if multiple subspecies were combined in the Pacific sampling, this would not diminish the strong isolation and diagnosable differences between the Pacific and North Atlantic populations.

### 4.5 High Genetic Diversity but Widespread Inbreeding Among Blue Whales

Consistent with previous studies, all blue whale populations were characterised by high levels of genome‐wide heterozygosity when compared to other baleen whales (Árnason et al. 2018; Jossey et al. 2024; Wolf et al. 2022) and other mammals (Brüniche‐Olsen et al. 2018; Palkopoulou et al. 2015). Although it may seem contradictory because of the decimation of blue whales during the industrial whaling period, the onset of this bottleneck occurred only two to three generations ago (Tønnessen and Johnsen 1982). This limited time frame makes it unlikely for such effects to be reflected in the genotype, due to insufficient time for genetic drift and recombination events at reduced N_e_ (Baker et al. 1993; Sremba et al. 2012; Wolf et al. 2022). Instead, other factors like population size, physiological parameters and the long‐term demographic history are more likely to determine the levels of heterozygosity seen today (Brüniche‐Olsen, Kellner, and DeWoody 2019; Charlesworth and Jensen 2022). Like proposed previously (Wolf et al. 2022), other measures should be consulted to evaluate the negative genomic impact of whaling on baleen whale populations.

A test for evolutionary neutrality in the three blue whale populations resulted in positive Tajima's D statistics that suggests either balancing selection actively maintaining multiple alleles or a recent population contraction (Schmidt and Pool 2002). The higher magnitude of these values contrasts with the slightly positive or neutral values reported in comparable studies of other baleen whale populations, such as fin whales and North Atlantic right whales (Crossman, Fontaine, and Frasier 2023; Wolf et al. 2022). However, the demographic models developed in those studies suggest either less severe declines (Wolf et al. 2022) or more prolonged and gradual population decreases (Crossman, Fontaine, and Frasier 2023). To determine whether a strong population contraction may have contributed to these values in blue whales, future studies will require demographic models based on larger sample sizes (e.g., more than 10 individuals, as required by stairwayplot2, Liu and Fu 2020).

Signs of inbreeding were found in all individuals except some highly heterozygous outliers. Although there were no major differences between the populations, these results highly diverge from comparable analyses done on an Icelandic fin whale population (Wolf et al. 2022). Comparing differences between runs of homozygosity results are often problematic due to many variable factors induced by different software, definition settings and reference genome qualities (Prasad, Lorenzen, and Westbury 2022). However, in this case, both analyses were done with comparable data, identical methods and both identified ROH by manual verification. Thus, the nearly 10‐fold higher inbreeding factors in the three blue whale populations compared to the Icelandic fin whale population, may indeed indicate increased inbreeding in all blue whale populations. These results are congruent with reports of a more impactful population decline of blue whales by whaling compared to fin whales (Tønnessen and Johnsen 1982).

While blue whales populations appear to be recovering, as indicated by recent IUCN reports (Cooke 2018; Weir 2023) and models (Monnahan, Branch, and Punt 2014), the genome‐wide effects observed here must be closely monitored to track the impact of the bottleneck and support continued recovery. Particularly, inbreeding depression may have potentially long‐term effects on the overall fitness of the species as shown by the results presented here. To prevent this down‐spiralling effect from gaining impactful momentum, it may be beneficial for the health of the populations to facilitate migration by establishing corridors of restricted shipping traffic. To identify migration routes that are worth protecting, migration modelling like already done on a smaller scale (Bedriñana‐Romano et al. 2021) and future population genomic studies like done here are needed to develop precise conservation plans in the future.

## 5 Conclusion

Genomic data from Northern Hemisphere blue whale populations revealed high levels of isolation and diagnosable, albeit lower, genomic differentiation between them, consistent with findings in the Western Australian pygmy blue whale. Therefore, it is proposed to classify North Atlantic and North Pacific blue whales as two distinct subspecies (*B. m. musculus* and *B. m. sulfureus*). The impact of whaling has led to a high frequency of inbreeding within blue whales and to a loss of rare genetic alleles, demonstrating the need for further conservation efforts to protect this iconic species. The combination of a taxonomic reassessment together with a population viability analysis may also serve as an example for future conservation genetic studies that aim to provide insight into the conservation of highly mobile or inaccessible populations.

## Author Contributions

M.W. and A.J. conceived and designed the study, M.W., M.J.J. and A.J. wrote the manuscript, M.W. conducted the analyses, M.J.J. aided with the computational analyses of the re‐sequencing data.

## Conflicts of Interest

The authors declare no conflicts of interest.

## Benefit‐Sharing

Benefits from this research accrue from providing scientific information relevant to conservation and sustainable use of biological diversity as well as by sharing of our data and results on public databases as described above.

## Supporting information


Data S1.

## Data Availability

Raw sequencing reads of blue whales have been deposited at the National Center for Biotechnology Information under the BioProject PRJNA955240. Raw short read data for the single sei whale individual can be found under the BioProject PRJNA957797. Most of the code used to generate the results presented can be found on GitHub: /mag‐wolf/RESEQ‐to‐Popanalyses/ and Zenodo: https://doi.org/10.5281/zenodo.7863314. All secondary data, namely the SNP datasets are uploaded to a Dryad repository: [dataset] (Wolf, de Jong, and Janke 2024). All other data needed to evaluate the conclusions of the paper are present in the paper and/or the Supporting Informations. Additional data related to this paper can be requested from the authors.
