# Supplementary material for: Ocean‐Wide Conservation Genomics of Blue Whales Suggest New Northern Hemisphere Subspecies
Source: Mol Ecol. 2024 Dec 17;34(2):e17619. doi: 10.1111/mec.17619 (PMC11701878; doi:10.1111/mec.17619)
Supplement: Supplementary file 1 — Data S1. [file MEC-34-e17619-s001.pdf]

# Supplementary Materials for

# Ocean-wide conservation genomics of blue whales suggest new Northern Hemisphere subspecies.

Magnus Wolf, Menno J. de Jong, Axel Janke

*Corresponding author: Magnus Wolf; Email: Magnus.Wolf@uni-muenster.de

**This PDF file includes:**

Fig. S1 to S5

- **Fig. S1** Geographic distribution of sampling locations of blue whale individuals on a traditional map.
- **Fig. S2** Determination of the optimal K value for the admixture-like analysis performed with the LEA package.
- **Fig. S3** Heterozygosity distribution across the randomly chosen Super_scaffold_9 depicting 20kbp long sliding windows.
- **Fig. S4** Comparison of mitogenomic differentiation statistics between blue whale subspecies reported in Morin et al. (2023) and the here tested populations.

Tab. S1 to S4

- **Tab. S1** General information for samples sequenced in this study.
- **Tab. S2** General information for samples retrieved for the population genetic analyses from the NCBI sequence read archive (SRA).
- **Tab. S3** Sequencing and mapping statistics for all sequenced and publicly available samples.
- **Tab. S4** F-statistic inference for f2 and f3 statistics calculated after Patterson et al. 2012.
- **Tab. S5** General information for samples retrieved for the dated phylogeny from NCBI SRA.

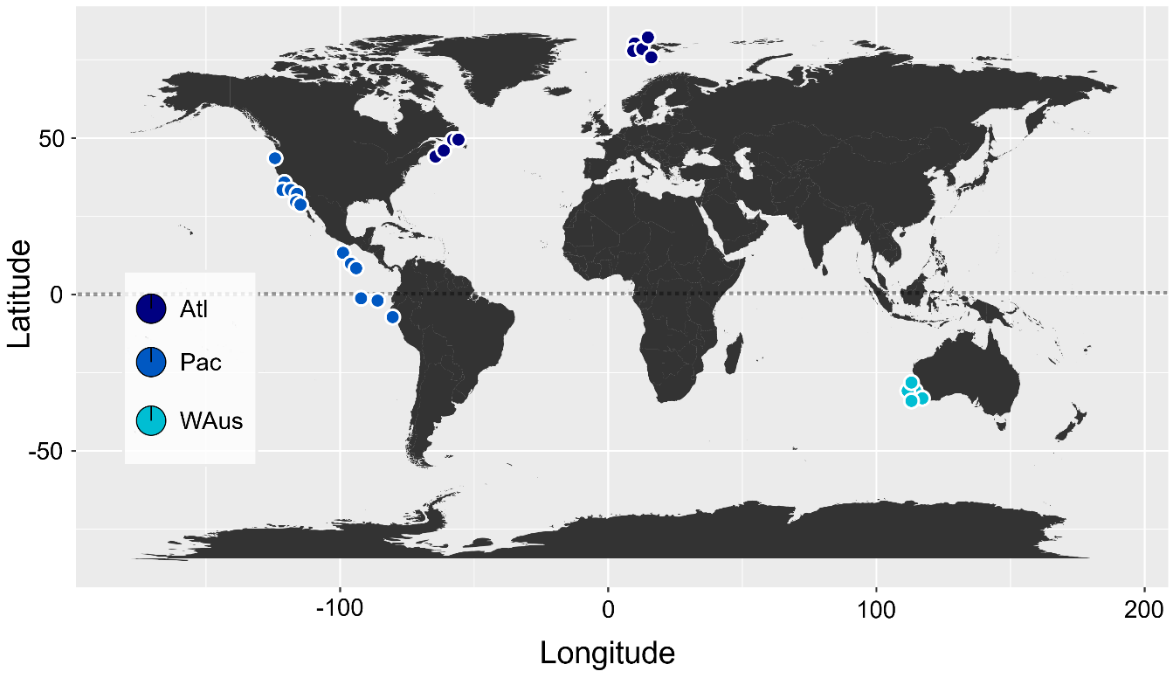


**Fig. S1** Geographic distribution of sampling locations of blue whale individuals on a traditional map. Colors were assigned to the respective oceanic region: dark blue: North Atlantic (Atl); light blue: Pacific (Pac); cyan: Western Australia (WAus). Dots indicate the specific location of the sample taken. The equator is indicated with a dotted line.

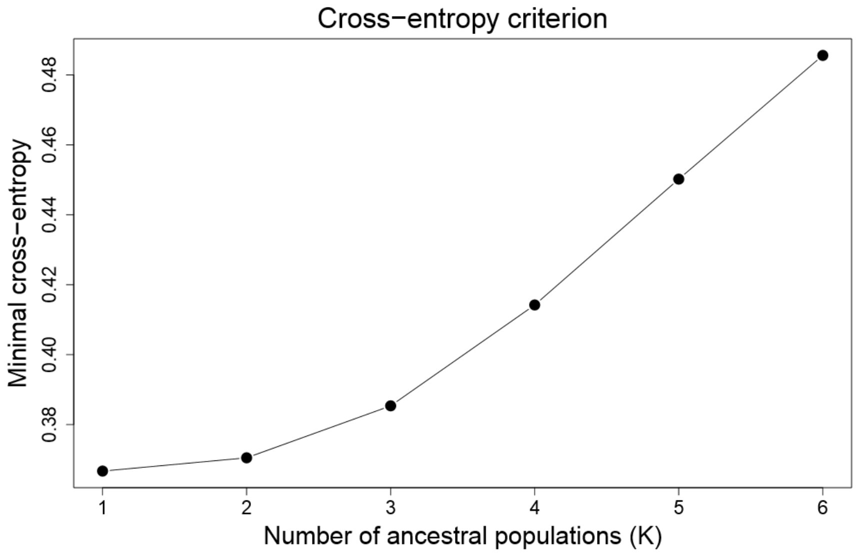


**Fig. S2** Determination of the optimal K value for the admixture-like analysis performed with the LEA-2.4.0 package (Frichot and François 2015). Shown are minimal cross-entropy scores generated with LEA's 'snmf' function.

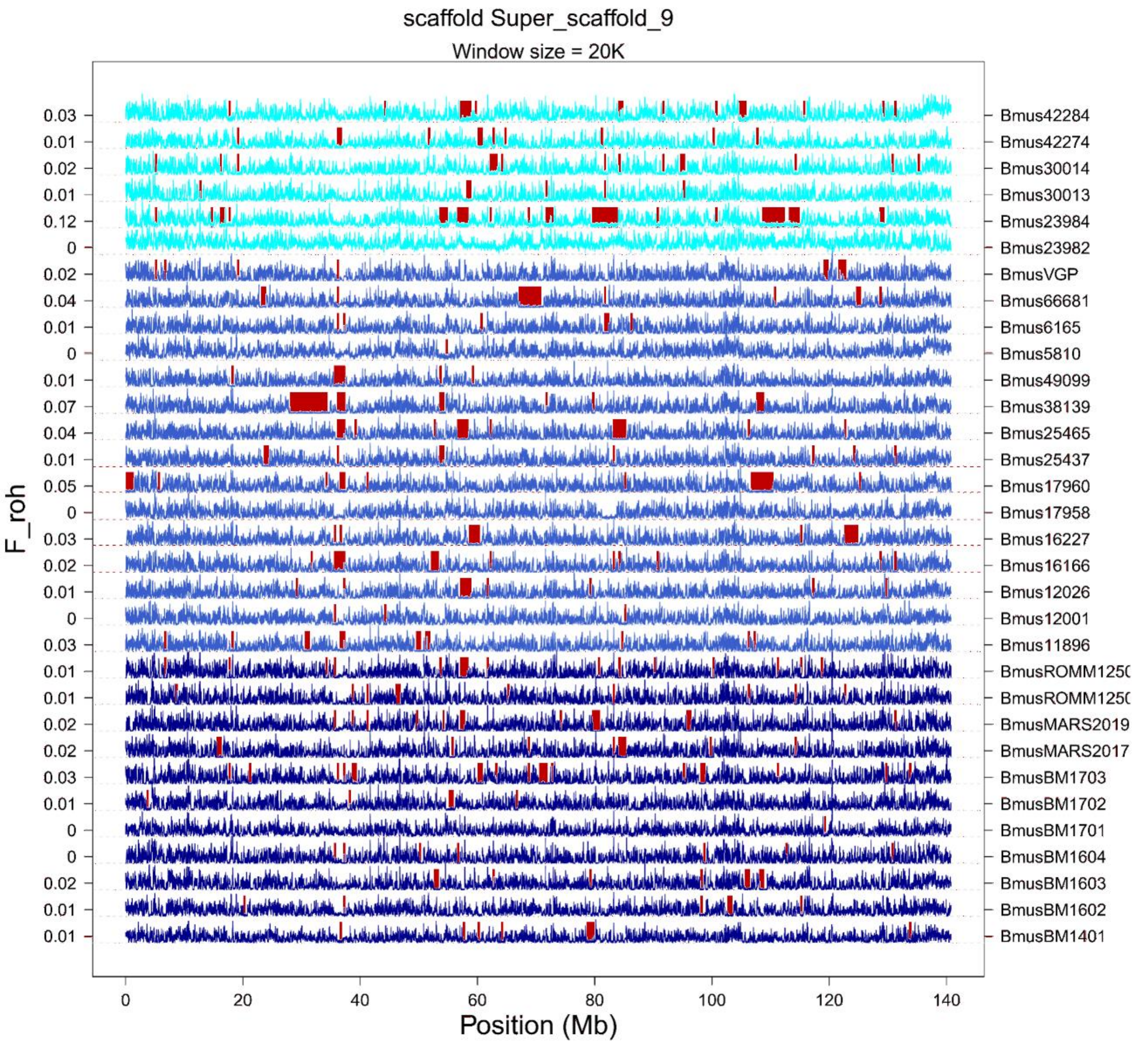


**Fig. S3** Heterozygosity distribution across the randomly chosen Super_scaffold_9 depicting 20 kbp long sliding windows. Runs of homozygosity found by DARWINDOW were highlighted in red and fit the visible drops in heterozygosity. Blue colors represent respective sampling locality: dark blue: Atl; light blue: Pac; cyan: WAus.

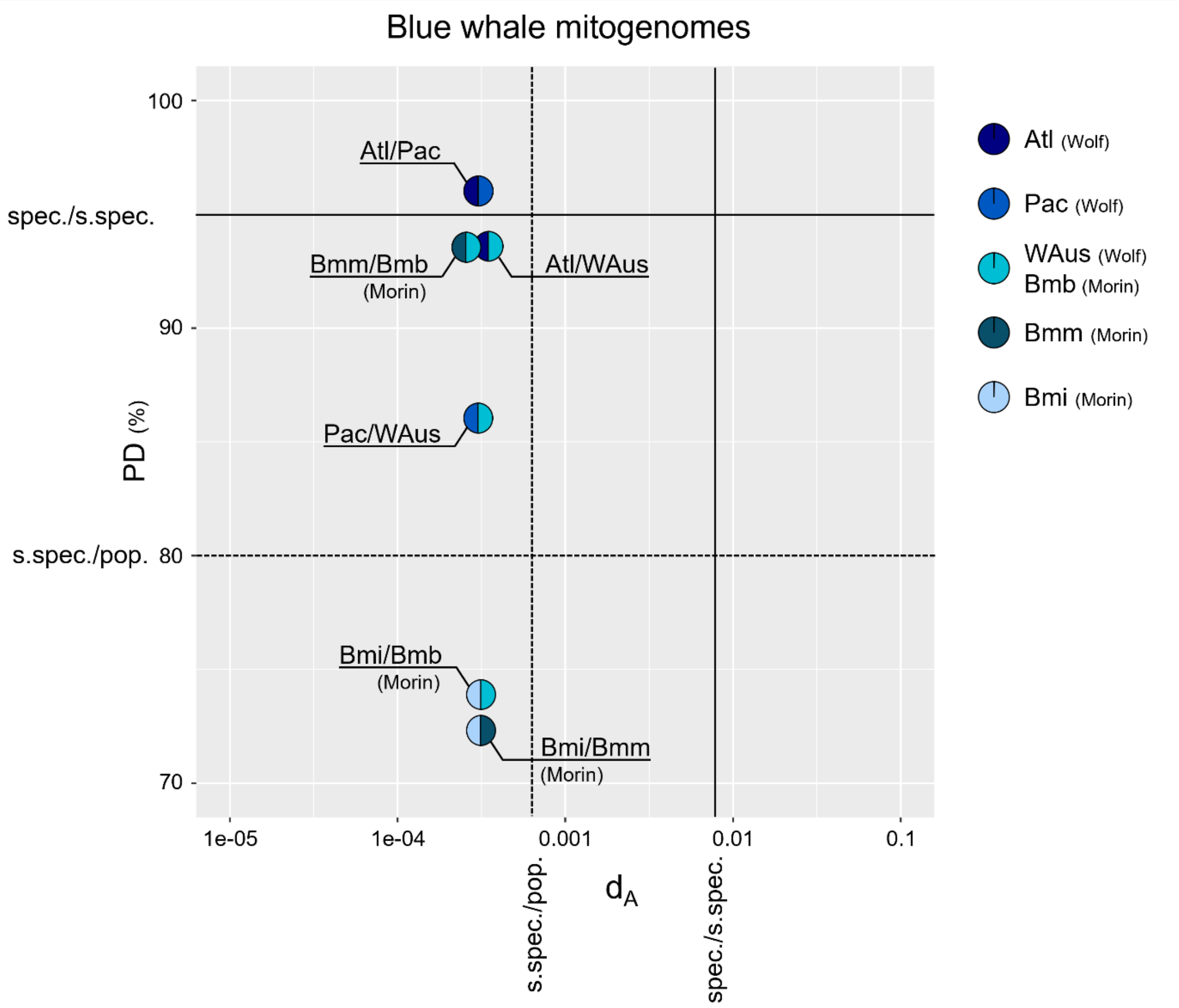


**Fig. S4** Comparison of mitogenomic differentiation statistics between blue whale subspecies reported in Morin et al. (2023) and the here tested populations. The x-axis depicts net nucleotide divergence ($d_A$) and the y-axis percent diagnosability (PD). Circles show pairwise comparisons of these statistics between either the populations from this study (Atl = North Atlantic, Pac = Pacific, WAus = Western Australia) or the already recognized subspecies reported in Morin et al. (2023) (Bmm = *B. m. musculus* / Northern Hemisphere, Bmb = *B. m. brevicauda* / Pygmy blue whale, Bmi = *B. m. intermedia* / Antarctic). Dashed lines indicate the population/subspecies threshold for these statistics proposed in Morin et al. (2023), full lines the subspecies/species threshold. The context provided by the recognized subspecies showcases that the Northern Hemisphere populations (Atl and Pac) feature similar and even more diagnosable differences between each other compared to already recognized subspecies pairs.

**Tab. S1** General information for samples sequenced in this study. Provided are field-IDs and source codes from the SWFSC collection as well as sampling date, location and sex. For the single sei whale, a lab ID is provided instead. Colors represent respective sampling locality or species: light blue: Pac; cyan: WAus; red: Sei whale.

| SWFSC ID | Field ID | SRA ID | Year | Month | Day | Latitude | Longitude | Sex |
|---|---|---|---|---|---|---|---|---|
| 5810 | DSJ960728.02 | SRR24296621 | 1996 | 7 | 28 | 34.15 | -120.283333 | F |
| 6165 | DSJ960908.02 | SRR24296622 | 1996 | 9 | 8 | 33.85 | -121.433333 | M |
| 11896 | END981127.02 | SRR24185311 | 1998 | 11 | 27 | -7.133333 | -80.583333 | M |
| 12001 | DSJ981106.01 | SRR24185303 | 1998 | 11 | 6 | -1.3 | -92.316666 | F |
| 12026 | DSJ981109.01 | SRR24185302 | 1998 | 11 | 9 | -1.833333 | -86.083333 | F |
| 16166 | DSJ991124.05 | SRR24185315 | 1999 | 11 | 24 | 8.35 | -93.95 | M |
| 16227 | MAC991016.04 | SRR24185312 | 1999 | 10 | 16 | 9.883333 | -96.016666 | F |
| 17958 | DSJ000730.04 | SRR24185314 | 2000 | 7 | 30 | 29.716666 | -115.816666 | F |
| 17960 | DSJ000730.06 | SRR24185313 | 2000 | 7 | 30 | 29.716666 | -115.816666 | M |
| 25437 | DSJ010921.12 | SRR24205218 | 2001 | 9 | 21 | 43.5 | -124.45 | F |
| 25465 | DSJ010930.12 | SRR24205221 | 2001 | 9 | 30 | 34.65 | -121.05 | F |
| 38139 | DSJ030921.04 | SRR24229944 | 2003 | 9 | 21 | 13.166666 | -99.05 | F |
| 49099 | HYDE051013.01 | SRR24230224 | 2005 | 10 | 13 | 32.85 | -117.316666 | - |
| 66681 | DSJ060730.11 | SRR24229238 | 2006 | 7 | 30 | 30.366666 | -116.433333 | F |
| 23982 | BMUS000328.01 | SRR24185316 | 2000 | 3 | 28 | -32.05 | 115.05 | M |
| 23984 | BMUS000229.01 | SRR24205217 | 2000 | 2 | 29 | NULL | NULL | F |
| 30013 | BM020130.01 | SRR24205222 | 2002 | 1 | 30 | -31.916666 | 114.966666 | M |
| 30014 | BM020319.01 | SRR24205116 | 2002 | 3 | 19 | -31.916666 | 115 | M |
| 42274 | BMUS-040207-6 | SRR24230178 | 2004 | 2 | 7 | -31.883333 | 115.033333 | F |
| 42284 | BMUS-040321-16 | SRR24229942 | 2004 | 3 | 21 | -31.016666 | 115.166666 | M |

| Lab ID | Source | SRA ID | Year | Month | Day | Latitude | Longitude | Sex |
|---|---|---|---|---|---|---|---|---|
| 8044 | CELL-CULTURE, ICELAND | SRR24229864 | 1986 | n.a. | n.a. | n.a. | n.a. | M |

**Tab. S2** General information for samples retrieved for the population genetic analyses from the NCBI sequence read archive (SRA). Samples are sorted by the BioProject. Provided are the species name and the respective study, the sample ID provided by the submitting authors, BioSample and SRA accession numbers as well as location and sampling years if available. Colors represent respective sampling locality or species: dark blue: Atl; red: Sei whale.

| | **BioProject: PRJNA704862** | | | | |
|---|---|---|---|---|---|
| **Species, Study** | **ID** | **Biosample** | **SRA** | **Location** | **Year** |
| *B. musculus*, Jossey et al. (2024) | **BM1703** | SAMN18057126 | SRR14357022 | 79.92 N 14.45 E | 2017 |
| | **BM1702** | SAMN18057125 | SRR14357025 | 78.73 N 9.26 E | 2017 |
| | **BM1701** | SAMN18057124 | SRR14357028 | 79.77 N 9.47 E | 2017 |
| | **BM1604** | SAMN18057123 | SRR14357029 | 78.45 N 12.15 E | 2016 |
| | **BM1603** | SAMN18057122 | SRR14357030 | 78.33 N 12.13 E | 2016 |
| | **BM1602** | SAMN18057121 | SRR14357032 | 78.33 N 12.13 E | 2016 |
| | **BM1401** | SAMN18057120 | SRR14357033 | Svalbard | 2014 |
| | **MARS2019313** | SAMN18057109 | SRR14357010 | Canada: Sutherlands Cove, Cape Breton Island, Nova Scotia | 2019 |
| | **MARS2017066** | SAMN18057108 | SRR14357011 | Canada: Ragged Harbour, Nova Scotia | 2017 |
| | **ROMM125065** | SAMN18057107 | SRR14357035 | Canada: Trout River, Newfoundland and Labrador | 2014 |
| | **ROMM125066** | SAMN18057106 | SRR14467046 | Canada: Rocky Harbour, Newfoundland and Labrador | 2014 |
| Bukhman et al. 2024 | **VGP** | SAMN12287136 | *VGP reposit.* | 34.42 N 119.7 W | 2016 |
| | **BioProject: PRJNA389516** | | | | |
| *B. borealis*, (Árnason et al. 2018) | **E91** | SAMN07201757 | SRS2268081 | Iceland | - |
| | **D27** | SAMN07201756 | SRS2268080 | Iceland | - |

**Tab. S3** Sequencing and mapping statistics for all sequenced and publicly available samples. Most statistics were collected with QUALIMAP v.2.2.2 (Okonechnikov et al. 2016). Provided are: the sample ID, the retrieved number of reads, the percentage of mapped reads, the estimated number of duplicated reads, mean insert sizes, mean mapping quality, the general error rate, the mean coverage, the coverage standard deviation, the number of retrieved SNPs after individual variant calling and filtering as described in the general method section. Colors represent respective sampling locality or species: dark blue: Atl; light blue: Pac; cyan: WAus; red: Sei whale.

| ID | No. reads | map. (%) | No. dup. | insert size | map. qual. | error rate | cov. | std cov. | No. Genotypes |
|---|---|---|---|---|---|---|---|---|---|
| 8044 | 1002713262 | 67.31 | 333507078 | 325498.8 | 52.3 | 0.0255 | 36.6 | 1097.4 | 1074255177 |
| D27 | 314819542 | 58.62 | 51853177 | 328485.2 | 52.3 | 0.0226 | 6.5 | 165.9 | 1185713484 |
| E91 | 311030310 | 56.23 | 50049832 | 335987.5 | 52.3 | 0.0230 | 6.1 | 168.3 | 1166774568 |
| 11896 | 299065816 | 73.36 | 94776896 | 313260.4 | 52.8 | 0.0160 | 11.7 | 406.3 | 1120026172 |
| 12001 | 310066206 | 72.84 | 98107266 | 323390.0 | 52.7 | 0.0164 | 12.0 | 416.9 | 1135367675 |
| 12026 | 313119553 | 72.86 | 99054969 | 306384.3 | 52.8 | 0.0162 | 12.2 | 419.8 | 1136632704 |
| 16166 | 332865840 | 72.97 | 105526910 | 314721.1 | 52.9 | 0.0156 | 13.0 | 451.9 | 1144583041 |
| 16227 | 342747354 | 72.58 | 106550949 | 318051.2 | 52.7 | 0.0157 | 13.3 | 427.2 | 1162833259 |
| 17958 | 319352518 | 74.46 | 105301016 | 310535.6 | 52.6 | 0.0161 | 12.5 | 415.9 | 1145284797 |
| 17960 | 303884812 | 73.44 | 96515165 | 313731.3 | 52.8 | 0.0168 | 11.8 | 398.8 | 1128455627 |
| 25437 | 309692751 | 72.81 | 96265851 | 303854.6 | 52.9 | 0.0155 | 12.1 | 410.7 | 1137371157 |
| 25465 | 314528954 | 73.08 | 97798675 | 315387.5 | 52.8 | 0.0158 | 12.3 | 403.0 | 1145343139 |
| 38139 | 308440586 | 73.27 | 97810167 | 303297.7 | 52.6 | 0.0157 | 12.0 | 383.7 | 1204192598 |
| 49099 | 301783257 | 77.06 | 114235174 | 322581.2 | 52.5 | 0.0149 | 12.0 | 450.7 | 1019490010 |
| 5810 | 256981584 | 83.97 | 111269399 | 277750.1 | 51.6 | 0.0158 | 10.2 | 288.6 | 1145150464 |
| 6165 | 303229759 | 75.8 | 89779560 | 277088.6 | 53.5 | 0.0133 | 12.3 | 369.9 | 1137898476 |
| 66681 | 295380727 | 74.7 | 101034474 | 335820.6 | 52.6 | 0.0158 | 11.6 | 420.2 | 1091480040 |
| VGP | 792170642 | 82.22 | 358076506 | 149926.2 | 53.6 | 0.0079 | 27.3 | 443.7 | 1192022008 |
| 23982 | 326212734 | 77.61 | 116761510 | 275044.8 | 52.6 | 0.0178 | 13.0 | 419.9 | 1127080068 |
| 23984 | 297223179 | 73.21 | 96789490 | 308749.2 | 52.5 | 0.0167 | 11.4 | 382.8 | 1193112097 |
| 30013 | 356320848 | 73.33 | 114376438 | 307276.4 | 52.8 | 0.0157 | 13.8 | 434.0 | 1161396329 |
| 30014 | 332432229 | 74.39 | 106811158 | 295282.8 | 52.8 | 0.0157 | 13.0 | 400.6 | 1148686642 |
| 42274 | 202758526 | 70.7 | 72124619 | 367694.8 | 51.1 | 0.0173 | 7.1 | 278.2 | 1131981618 |
| 42284 | 283704417 | 74.82 | 98537906 | 284394.0 | 52.2 | 0.0170 | 10.8 | 341.8 | 1158089121 |
| BM1401 | 371281836 | 75.08 | 116530598 | 317297.2 | 52.9 | 0.0174 | 15.1 | 471.0 | 1185656170 |
| BM1602 | 267204560 | 72.94 | 80863656 | 323908.1 | 52.9 | 0.0186 | 10.6 | 358.3 | 1181598689 |
| BM1603 | 381649402 | 75.19 | 121328515 | 312444.8 | 53.0 | 0.0175 | 15.5 | 483.7 | 1187232718 |

| | | | | | | | | | | |
|---|---|---|---|---|---|---|---|---|---|---|
| **BM1604** | 789197681 | 74.7 | 272581539 | 335318.0 | 53.1 | 0.0170 | 31.8 | 932.5 | 1198204665 |
| **BM1701** | 164172844 | 73.71 | 48123384 | 321394.8 | 52.9 | 0.0190 | 6.5 | 227.6 | 1055275853 |
| **BM1702** | 348980624 | 75.49 | 112019450 | 323874.9 | 52.9 | 0.0176 | 14.2 | 478.2 | 1169554630 |
| **BM1703** | 373832583 | 75.84 | 118893261 | 312347.6 | 53.0 | 0.0173 | 15.3 | 470.9 | 1185651257 |
| **MARS2017066** | 628020829 | 68.3 | 197331572 | 286476.8 | 52.8 | 0.0201 | 23.0 | 717.3 | 1129916638 |
| **MARS2019313** | 718789733 | 72.17 | 240853247 | 338577.0 | 53.1 | 0.0181 | 28.3 | 894.0 | 1175537818 |
| **ROMM125065** | 792665299 | 73.04 | 280504311 | 341357.8 | 52.8 | 0.0161 | 31.5 | 992.9 | 1191966717 |
| **ROMM125066** | 805021222 | 72.37 | 272981880 | 347630.4 | 53.0 | 0.0157 | 31.9 | 986.5 | 1195497540 |

**Tab. S4** F-statistic inference for f2 and f3 statistics calculated after Patterson et al. (2012). **A** f2 matrix providing pairwise values. **B** f3 table providing triplet values between the tested population (color highlighted) and the two assumed ancestral populations. Table also includes standard errors, Z-scores and p-values. Colors represent respective sampling locality or species: dark blue: Atl; light blue: Pac; cyan: WAus; red: Sei whale.

**A f2 Statistics** after Patterson et al. (2012)

| f2 | Atl | Pac | WAus | Sei |
|---|---|---|---|---|
| **Atl** | 0 | 0.020 | 0.051 | 0.543 |
| **Pac** | 0.020 | 0 | 0.043 | 0.543 |
| **WAus** | 0.051 | 0.043 | 0 | 0.557 |
| **Sei** | 0.543 | 0.543 | 0.557 | 0 |

**B f3 Statistics** after Patterson et al. (2012)

| Test Pop1 | Anc. Pop2 | Anc. Pop3 | f3 | SE | Z-score | P |
|---|---|---|---|---|---|---|
| **Atl** | Pac | WAus | 0.0147 | 0.00015 | 97.48 | 0 |
| **Pac** | WAus | Atl | 0.0057 | 0.00013 | 45.72 | 0 |
| **WAus** | Atl | Pac | 0.0372 | 0.00027 | 135.48 | 0 |
| **Sei** | Atl | Pac | 0.5384 | 0.00072 | 774.44 | 0 |
| **Sei** | WAus | Atl | 0.5363 | 0.00073 | 735.09 | 0 |
| **Sei** | WAus | Pac | 0.5384 | 0.00073 | 741.82 | 0 |
| **Atl** | Sei | Pac | 0.0053 | 0.00008 | 61.31 | 0 |
| **Atl** | Sei | WAus | 0.0073 | 0.00013 | 57.52 | 0 |
| **Pac** | Sei | Atl | 0.0048 | 0.00008 | 58.64 | 0 |
| **Pac** | Sei | WAus | 0.005 | 0.00012 | 41.54 | 0 |
| **WAus** | Sei | Atl | 0.0189 | 0.00017 | 114.57 | 0 |
| **WAus** | Sei | Pac | 0.0168 | 0.00015 | 109.61 | 0 |

**Tab. S5** General information for samples retrieved for the dated phylogeny from NCBI SRA. Provided are the species name, the respective study, the BioProject, BioSample and SRA accession number. Red colors represent their position outside of the blue whale clade as depicted in Fig. 4 of the main document.

| *Species* | Study | BioProject | BioSample | SRA |
|---|---|---|---|---|
| *B. acutorostrata* | Yim et al. 2014 | PRJNA72723 | SAMN02192644 | SRR924087 |
| *Eschrichtius robustus* | Árnason et al. 2018 | PRJNA389516 | SAMN07201759 | SRR5665641 |
| *B. physalus* | Wolf et al. 2022 | PRJNA740292 | SAMN20858610 | SRR15525328 |
| *Megaptera novaeangliae* | Tollis et al. 2019 | PRJNA509641 | SAMN10585801 | SRR8385995 |
| *Eubalaena glacialis* | DNA Zoo | PRJNA512907 | SAMN14122067 | SRR11097130 |

## References


Árnason Ú, Lammers F, Kumar V, Nilsson MA, Janke A. 2018. Whole-genome sequencing of the blue whale and other rorquals finds signatures for introgressive gene flow. *Science advances*. 4(4):eaap9873.

Bukhman YV, Morin PA, Meyer S, et al. (33 co-authors). 2024. A High-Quality Blue Whale Genome, Segmental Duplications, and Historical Demography. Molecular biology and evolution. 41(3).

Frichot E, François O. 2015. LEA. An R package for landscape and ecological association studies. *Methods Ecol Evol*. 6(8):925–929.

Jossey S, Haddrath O, Loureiro L, et al. (10 co-authors). 2024. Population structure and history of North Atlantic Blue whales (Balaenoptera musculus musculus) inferred from whole genome sequence analysis. *Conserv Genet*. 25(2):357–371.

Morin PA, Martien KK, Lang AR, et al. (9 co-authors). 2023. Guidelines and quantitative standards for improved cetacean taxonomy using full mitochondrial genomes. The Journal of heredity. 114(6):612–624.

Okonechnikov K, Conesa A, García-Alcalde F. 2016. Qualimap 2: advanced multi-sample quality control for high-throughput sequencing data. *Bioinformatics (Oxford, England)*. 32(2):292–294.

Patterson N, Moorjani P, Luo Y, Mallick S, Rohland N, Zhan Y, Genschoreck T, Webster T, Reich D. 2012. Ancient admixture in human history. *Genetics*. 192(3):1065–1093.

Tollis M, Robbins J, Webb AE, et al. (9 co-authors). 2019. Return to the Sea, Get Huge, Beat Cancer: An Analysis of Cetacean Genomes Including an Assembly for the Humpback Whale (Megaptera novaeangliae). *Molecular biology and evolution*. 36(8):1746–1763.

Wolf M, Jong M de, Halldórsson SD, Árnason Ú, Janke A. 2022. Genomic Impact of Whaling in North Atlantic Fin Whales. *Molecular biology and evolution*. 39(5).

Yim H-S, Cho YS, Guang X, et al. (52 co-authors). 2014. Minke whale genome and aquatic adaptation in cetaceans. *Nature genetics*. 46(1):88–92.
